# Analysis of computer-aided diagnostics in the preoperative diagnosis of ovarian cancer: a systematic review

**DOI:** 10.1186/s13244-022-01345-x

**Published:** 2023-02-15

**Authors:** Anna H. Koch, Lara S. Jeelof, Caroline L. P. Muntinga, T. A. Gootzen, Nienke M. A. van de Kruis, Joost Nederend, Tim Boers, Fons van der Sommen, Jurgen M. J. Piek

**Affiliations:** 1 Department of Gynaecology and Obstetrics and Catharina Cancer Institute, Catharina Hospital, 5623 EJ Eindhoven, Noord-Brabant, The Netherlands; 2 Department of Radiology, Catharina Hospital, 5623 EJ Eindhoven, Noord-Brabant, The Netherlands; 3 Department of Electrical Engineering, VCA Group, University of Technology Eindhoven, 5600 MB Eindhoven, Noord-Brabant The Netherlands

**Keywords:** Diagnosis, Computer-assisted, Machine learning, Ovarian neoplasms

## Abstract

**Objectives:**

Different noninvasive imaging methods to predict the chance of malignancy of ovarian tumors are available. However, their predictive value is limited due to subjectivity of the reviewer. Therefore, more objective prediction models are needed. Computer-aided diagnostics (CAD) could be such a model, since it lacks bias that comes with currently used models. In this study, we evaluated the available data on CAD in predicting the chance of malignancy of ovarian tumors.

**Methods:**

We searched for all published studies investigating diagnostic accuracy of CAD based on ultrasound, CT and MRI in pre-surgical patients with an ovarian tumor compared to reference standards.

**Results:**

In thirty-one included studies, extracted features from three different imaging techniques were used in different mathematical models. All studies assessed CAD based on machine learning on ultrasound, CT scan and MRI scan images. Per imaging method, subsequently ultrasound, CT and MRI, sensitivities ranged from 40.3 to 100%; 84.6–100% and 66.7–100% and specificities ranged from 76.3–100%; 69–100% and 77.8–100%. Results could not be pooled, due to broad heterogeneity. Although the majority of studies report high performances, they are at considerable risk of overfitting due to the absence of an independent test set.

**Conclusion:**

Based on this literature review, different CAD for ultrasound, CT scans and MRI scans seem promising to aid physicians in assessing ovarian tumors through their objective and potentially cost-effective character. However, performance should be evaluated per imaging technique. Prospective and larger datasets with external validation are desired to make their results generalizable.

**Supplementary Information:**

The online version contains supplementary material available at 10.1186/s13244-022-01345-x.

## Key points

- Computer-aided diagnostics has potential to predict the nature of ovarian tumors.
- Literature shows heterogeneous sensitivity and specificity of machine learning on ultrasound images, CT-scan images and MRI-scan images.
- More prospective studies on other computer-aided techniques and imaging modalities should be performed with an external validation set.

**Supplementary Information:**

The online version contains supplementary material available at 10.1186/s13244-022-01345-x.

## Introduction

An accurate preoperative diagnosis of an ovarian tumor into either benign, borderline or malignant is important for multiple reasons: (1) for the patients’ surgical workup and treatment planning, (2) for the patients’ mental wellbeing and (3) for correct use of diagnostic algorithms [1]. Currently, most women diagnosed with an ovarian tumor are initially evaluated with transvaginal ultrasound and serum CA125. For a more objective approach, different ultrasound-based models, to discriminate between benign, borderline and malignant ovarian tumors, have been constructed over time. One of the first widely used models is the risk of malignancy index (RMI) which combines five ultrasound variables with serum CA125 and postmenopausal status [2]. Other models have been developed by the International Ovarian tumor analysis (IOTA) group, such as the Assessment of Different NEoplasias in the adneXa (ADNEX) model, which combines six ultrasound features together with patients age, serum CA125 and type of center (oncology referral center vs other) [3, 4]. However, for both models the reported sensitivity lies around 98% and 71%, and the specificity around 85% and 62% [5]. In addition, two other classification models were introduced by radiologists and gynecologists: (1) the GI-RADs (Gynecologic Imaging Reporting and Data System) score, for diagnosis of adnexal masses by pelvic ultrasound, and (2) the O-RADS (Ovarian-adnexal reporting and data system) data system, both showing a sensitivity of 92.7% and 93.6% and a specificity of 96.8% and 92.8% [6, 7]. Nevertheless, research has shown that ultrasound features are often misclassified by unexperienced examiners [8].

Nowadays, preoperative computer tomography (CT) and/or magnetic resonance imaging (MRI) is performed to pre-surgically assess the nature of an ovarian tumor and to predict the presence of metastatic disease. MRI has proven to be able to discriminate between benign and malignant ovarian tumors with a sensitivity of 96% and a specificity of 91%. The O-RADs MRI has a sensitivity of 93% and a specificity of 91% for score 5 (malignant) with a comparable reading between senior and junior radiologists [7, 9, 10]. However, for spiral CT scans no diagnostic studies are available. Research conducted with multidetector CT scans shows an accuracy of 90 to 93% in adnexal mass characterization [11].

For clinicians, ideally, when using any test a 100% sensitivity and specificity is desired. For imaging prediction models, this means that no malignant tumors are missed and no benign tumors are classified as malignant to prevent unnecessary surgical procedures on benign ovarian tumors [12, 13]. Hence, diagnostic accuracy with a higher sensitivity at the detriment of the specificity is favorable. The currently used imaging prediction models show high performance in ovarian tumor classification; nevertheless, they are greatly affected by subjective assessment and users’ experience. Therefore, evaluation of more independent strategies to determine the nature of ovarian tumors among these different imaging modalities is needed.

Over the past three decades, several computer-aided diagnostics (CADs) have been developed for accurate ovarian cancer prediction, mainly on ultrasound, all using predefined hand-selected features to build their classifiers [14–16]. Computer-aided diagnostics is used to assist clinical professionals within different medical specialties, such as dermatology, neurology and pathology [17–20]. Furthermore, it can aid radiologists’ image interpretations and extract features from medical images, which are not visible for the human eye, giving it a cost-effective potential as well [21]. Still, within the field of gynecologic oncology it is relatively new compared to other medical specialties [22].

In this study, we assess the available literature on CAD in preoperatively predicting the chance of an ovarian malignancy.

## Materials and methods

We searched for all published studies investigating diagnostic accuracy of CAD based on ultrasound, CT and MRI in patients with an ovarian tumor. Search terms used were: ‘ovaria,’ ‘ovarian neoplasms,’ ‘ovarian neoplasm,’ ‘ovarian masses,’ ‘ovarian lesion,’ ‘ovarian tumor,’ ‘adnexal,’ ‘adnexal mass,’ ‘ovarian cancer,’ ‘ovarian malignancy,’ ‘ovary,’ ‘classification of ovarian,’ ‘machine learning,’ ‘computer aided,’ ‘Diagnosis Computer-Assisted,’ ‘computer assisted-diagnosis,’ ‘artificial intelligence,’ ‘Neural Networks, Computer,’ ’convolutional neural network,’ ‘radiomics,’ ‘decision support system,’ ‘decision support technique,’ ‘decision support techniques,’ ‘machine learning classifier,’ ‘machine learning classifiers,’ ‘diagnosis,’ ‘diagnostic accuracy,’ ‘presurgical,’ ‘preoperative,’ ‘preoperative diagnosis,’ ‘preoperative evaluation,’ ‘Tomography, X-ray Computed,’ ‘ct-scan,’ ‘ultrasound,’ ‘echography,’ ‘gynecological ultrasound,’ ‘ultrasonography,’ ‘magnetic resonance imaging,’ ‘nuclear magnetic resonance imaging’ and ‘MRI.’ We used ‘title abstract’ (tiab) and ‘Mesh’ added to each search term. The exact search syntax per database is provided in Additional file 1: Appendix 1.

The search was last performed on 9th 2022 by two independent reviewers and a research librarian was consulted for support in this matter.

We searched for papers published in English in Cochrane Central Register of Controlled Trials, MEDLINE, Embase, Scopus and PubMed. Additionally, we searched trial registries for ongoing and registered trials on Clinicaltrials.gov. To identify additional trials, references of all included studies by the initial search were hand searched to add relevant trials.

All studies that investigated diagnostic accuracy of CAD based on ultrasound, CT and MRI images in patients with an adnexal mass were included. Case reports, summaries, animal studies, meta-analyses, comments, editorials, conference abstracts and other irrelevant article types were excluded.

### Selection of studies

**Fig. 1:**
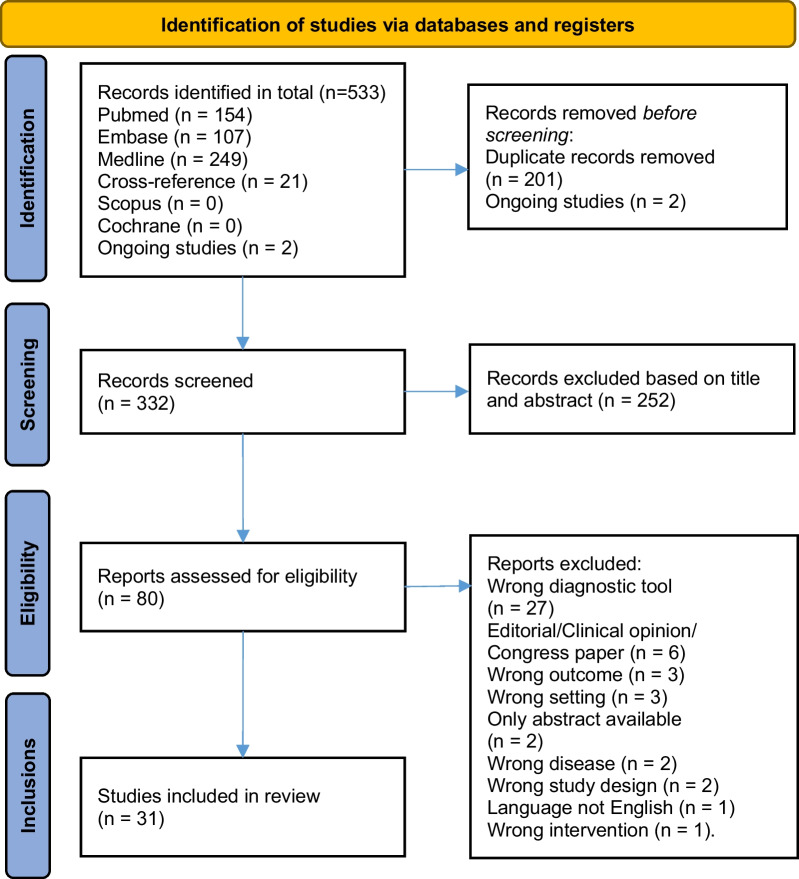
PRISMA flowchart [32]

Titles and abstracts retrieved by the search were imported into the reference manager database Covidence [23]. Duplicates were removed and two reviewers independently screened the records. Subsequently, full-text versions of potentially relevant studies were obtained and assessed for eligibility by the same researchers. Studies were qualified if the following criteria were met: (1) accurate disease type, e.g., benign, borderline or malignant ovarian tumors, (2) appropriate clinical setting, for example, no ex vivo studies, (3) description of overfitting techniques and reference standard, (4) use of correct classifier, e.g., none of the features selected to construct the CAD were manually measured, as done by Timmerman et al*.*, Biagiotti et al*.* or Zimmerman et al*.* [14–16] and (5) diagnostic accuracy had to be reported, namely sensitivity, specificity or area under the curve (AUC). Disagreements were resolved through discussion until consensus was reached, or by consulting a third member of the review team. The selection process was visualized in a PRISMA flowchart (Fig. 1).

### Data extraction and management

Two reviewers independently extracted the following data from each included study: study design, year of publication, country where the study was conducted, inclusion and exclusion criteria or population description, number of participants, menopausal status, mean CA125 serum levels of included participants, number of images, intervention compared to histology, type of classifier and features used to develop the CAD, duration of follow-up, reference standard and results. When multiple classifiers were described, the best performing one was selected. Supplementary appendices were assessed for additional study details and corresponding authors were contacted by email on study details if necessary. Discrepancies were resolved through discussion and consensus, or by consulting a third member of the review team. Study outcomes were type of classifier, whether an external validation set was used, if CAD was compared to or combined with other models or subjective assessment (SA), sensitivity, specificity, accuracy and AUC, when mentioned in the included study. Other diagnostic accuracy values were also considered. We aimed to perform a meta-analysis of the CAD methods that used an external validation set, for which *Review Manager* (RevMan) software (v5.4.1) and Meta-DiSc software were utilized [24]. Heterogeneity was assessed by using the I2 statistics, which describes the percentage of variability due directly to heterogeneity, with > 50% representing moderate heterogeneity and > 75% indicating high heterogeneity and Moses-Littenberg SROC (summary receiver operating curve) plot [25, 26].

### Assessment of risk of bias of included studies

Two independent reviewers assessed the methodological quality of each included study by using the Prediction Model Study Risk of Bias Assessment Tool (PROBAST) together with additional questions from the quality assessment of diagnosis accuracy study (QUADAS-2) tool and the quality in prognostic studies (QUIPS) tool. Discrepancies were resolved through discussion and consensus, or by consulting a third member of the review team. Different risk of bias assessment tools were used because different types of study designs were included. Studies that evaluated multivariable diagnostic or prognostic prediction models were reviewed using PROBAST. PROBAST assesses four key domains: participants, predictors, outcome and analysis. Studies that evaluated diagnostic tests of prognostic factors were reviewed by a few questions from the QUADAS-2 tool and the QUIPS tool [27–30]. Furthermore, seven signaling questions were composed by independent technical members of the study team to assess risk of bias based on the used CAD model, called ‘CAD model risk of bias screening questions.’ These two members were not aware of the content of the articles included. These signaling questions are described in Additional file 1: Appendix 2.

The signaling questions were used to determine whether risk of bias was low, high or unclear.

The extraction of study data, comparisons in data tables and preparation of a ‘Summary of findings’ table were performed before writing the results and conclusions of this review.

The protocol of this systematic review was registered with PROSPERO (Registration number CRD42020189910).

## Results

After the search was performed and cross-reference articles were added, a total of 532 articles were retrieved. Subsequently, duplicates were removed and 331 articles remained for screening on title and abstract. Seventy-one articles were eligible for full-text reading. Two studies on CAD and ovarian tumors were found on ClinicalTrial.gov. Both trials are open for accrual and are using CAD in diagnosing (1) malignant ovarian tumor with CT (NCT05174377) and (2) endometriosis-related ovarian cancer (NCT05161949). Most articles were excluded because they were not using CAD, not assessing ovarian tumors or because a wrong type of classifier was used. A summary of the selection process is shown in a PRISMA flowchart (Fig. 1) [31, 32].

After screening the title, abstract and full-text thirty-one studies were included in this systematic review.

### Description of included studies

Thirty-one studies were included in this review, of which twenty-two ultrasound-based studies [33–53, 62], three CT-based studies [54–56] and six MRI-based studies [57–62]. A detailed overview of the included studies is presented in Additional file 2: Table 1a–c. There were twenty-two retrospective studies of which nineteen are case–control studies and two are cohort studies. Six studies have a prospective case–control design, and one is a cohort study. Women of all ages were included in the studies. Only seven studies used external validation datasets to assess the performance of their classifier: four ultrasound, one CT and two MRI studies [33, 35, 51, 53, 56, 59, 61]. The same dataset was used in ten studies to develop and test different classifiers [43, 45, 49, 50, 52, 54, 56, 59, 61, 62]. In most studies, the region of interest (ROI) was annotated manually. Two studies did not mention histology as definite diagnosis [41, 42]. Only three studies combined CAD with clinical features. Eleven studies compared the CAD with subjective assessment (SA) of a reviewer or combined the CAD model with SA performance of the reviewer [33, 35–38, 48, 50, 58, 60–62]. Table 1a–c presents the results of each study.

**Table 1:** Results depicted per image modality: ultrasound, CT and MRI

Included studies	Study setting	Patients (n)	Samples (n)	CAD-model	Features (n)	Performance ACC	Performance AUC	Performance Sensitivity	Performance Specificity	Performance Other	CAD model evaluation method	Compared to other models or reviewer(s)*
*a: CAD ultrasound (22)*												
Gao et al*.* [33]	Retrospective Case–control	107,624	575,930 images	DCNN	121 layers	(1) 86.9%	(1) 0.870)	(1) 40.3%	(1) 91.6%	Brier-score	1 internal validation set	Radiologist alone (3) Radiologist with DCNN (4)
			103,370 benign			(2) 85.3%	(2) 0.831	(2) 57.8%	(2) 98.5%	F1-score	2 external validation set (1 + 2)	
			4254 malignant			(3) 81.1%	(3) N/A	(3) 55.5%	(3) 87.5%	PPV		
						(4) 87.6%	(4) N/A	(4) 82.7%	(4) 88.7%	NPV		
Chiappa et al*.* [34]	Retrospective Case–control	241	241 images	SVM	853	80.00%	0.83	78.00%	83.00%	N/A	Training-Validation Testing Nested-tenfold validation	N/A
			115 benign		269 solid	87.00%	0.88	75.00%	90.00%			
			126 malignant		278 cystic	81.00%	0.89	81.00%	81.00%			
					306 motley							
Chiappa et al*.* [35]	Retrospective & Prospective Case–control	274	274 images	DSS	857	(1) 87.9%	N/A	(1) 99.2%	(1) 75.9%	PPV	External validation in prospective cohort (n = 35) tenfold cross validation	2 gynecologists with DCNN (1 + 2) on internal & external dataset
		239	239		269 solid	(2) 88.7%		(2) 98.4%	(2) 78.5%	NPV		
		35	123 benign		278 cystic	(1) 91.4%		(1) 100.0%	(1) 80.0%			
			116 malignant		306 motley	(2) 91.4%		(2) 100.0%	(2) 80.0%			
			35		4 clinical							
			15 benign									
			20 malignant									
Christiansen et al. [36]	Retrospective Case–control	758	3077	VGG16	1024 layers	91.30%	0.95	96.00%	86.70%	N/A	Training 67%	SA (2)
		634 surgery	1927 grayscale	ResNet	512 layers	(2) 92.0%	N/A	(2) 96.0%	(2) 88.0%		Validation 13%	RMI (3)
		124 follow-up	1150 power doppler	MobileNet		(3) 93.6%		(3) 94.5%	(3) 92.6%		Testing 20%	SR (4)
				(Ovry-Dx1)		(4) 96.0%		(4) 66.7%	(4) 81.3%			SRR (5)
			449 benign									
			309 malignant									
Qi et al*.* [37]	Retrospective Case–control	265	279 images	Nomogram with LASSO and RADscore	17	(1) 88.0%	(1) 0.914	(1) 81.3%	(1) 92.2%	IDI	Training 70% Validation 30% tenfold cross validation	Senior (3) & Junior (4) sonographists
			106 benign	task 1 + 2	22	(2) 86.3%	(2) 0.890	(2) 84.2%	(2) 97.5%			task 1 benign – malignant (1)
			65 borderline tumors		4 clinical	(3) 79.5%	(3) 0.789	(3) 69.7%	(3) 86.0%			task 2 benign-borderline-malignant (2)
			108 malignant tumors			(3) 64.7%	(3) 0.612	(3) 53.6%	(3) 68.6%			
						(4) 69.9%	(4) 0.669	(4) 56.8%	(4) 80.4%			
						(4) 56.9%	(4) 0.521	(4) 56.3%	(4) 62.2%			
Stefan et al. [63]	Retrospective Case–control	120	123 images	KNN	3	85.37%	N/A	80.00%	87.50%	PPV	Run KNN twice**	N/A
			85 benign									
			35 malignant									
Wang et al*.* [38]	Retrospective Case–control	265	279 images 108 benign	VGG	N/A	(1) 91.4%	(1) 0.963	(1) 91.4%	(1) 91.4%	F1-score	Transfer learning	Sonographist (3) task C (benign-borderline-malignant)
			65 borderline 106 malignant	GoogleNet		(2) 75.3%	(2) N/A	(2) 80.0% / 45.5% / 88.9%	(2) 89.7% / 95.8% / 75.4%		threefold-cross validation	task A benign – malignant (1)
				**ResNet**		(3) 66.7%	(3) N/A	(3) 75.0% / 47.4% / 68.4%	(3) 81.8% / 85.2% / 82.5%			
				MobileNet								
				task A + C (1) + (2)								
Martinez-Mas et al. [39]	Retrospective Case–control	187	384 images 112 benign	SVM	N/A	87.70%	0.874	92.00%	80.00%	N/A	LOO-CV	N/A
			75 malignant	KNN							N = 30	
				LD								
				**ELM**								
Zhang et al. [40]	Retrospective Case–control	N/A	428 images 357 malignant 71 benign 1400 images 277 malignant 299 benign	**Cost-sensitive RF**	N/A	99.20%	0.997	99.70%	95.60%	N/A	Transfer learning	N/A
				VGGNet							Training 71.5%	
				GoogleNet							Validation 14.3%	
				FCNN							Testing 14.3%	
				AlexNet							tenfold-cross validation	
Acharya et al*.* [41]	N/A	469	469	KNN	39	80.60%	0.806	81.40%	76.30%	N/A	tenfold cross validation	N/A
	Cohort		238 suspicious 281 non-suspicious	RF								
				**FF**								
				FRNN								
Aramendia-Vidaurreta et al*.* [46]	N/A	145	145 images 106 benign	MLP	40	98.80%	0.997	98.50%	98.90%	PPV	Training 80% Validation 10% Testing 10%	N/A
	Case–control		39 malignant		1 clinical						tenfold cross validation	
											40:30:01	
Khazendar et al*.* [47]	Retrospective Cohort	177	187 images	SVM	1	78.00%	N/A	80.00%	77.00%	T-test	Training and testing set	N/A
			112 benign	LBP on enhanced image							50-fold cross validation Performance of the SVM per 15 cycles	
			75 malignant									
Acharya et al*.* *** [44]	Retrospective Case–control	20	10 benign	SVM	11	(1) 100%	N/A	(1) 100%	(1) 100%	N/A	Training and testing set	N/A
			10 malignant 2600 images 1300 benign 1300 malignant	**KNN**		(2) 100%		(2) 100%	(2) 100%		tenfold cross validation	
				**PNN**								
Acharya et al*.* **** [42]	Prospective cohort	23	20	PNN	23	99.81%	N/A	99.92%	99.69%	PPV	Training 90%	N/A
			10 benign								Testing 10%	
			10 malignant								tenfold-cross validation	
			2600 images									
			1300 benign									
			1300 malignant									
Acharya et al*.* [45]	Prospective Case–control	10	20	DT	4	N/A	N/A	94.30%	99.70%	PPV	Training and testing set	N/A
			10 benign							TP rate	tenfold cross validation	
			10 malignant							FP rate		
										TN rate		
			2000 images							FN rate		
			1000 benign									
			1000 malignant									
Faschingbauer et al*.* [48]	Retrospective Case–control	105	105	SVM-ABTA	(1) 16	(1) N/A	N/A	(1) 69%	(1) 86%	Youden-index	Training and testing set	Level III gynaecologists (5)
			70 benign	Malignant (1)	(2) 16	(2) N/A		(2) 72%	(2) 81%		onefold cross validation	
			35 malignant	Dermoid cysts (2)	(3) 16	(3) N/A		(3) 82%	(3) 96%			
				Functional cysts (3) Overall (4)	(4) 16	(4) 74.3%		(4) N/A	(4) N/A			
						(5) 83.75%						
Acharya et al*.* [43]	Retrospective Cohort	20	20	SVM-RBF	14	99.90%	N/A	100%	99.80%	PPV	Training and testing set	N/A
			10 benign							TP rate	tenfold cross validation	
			10 malignant							FP rate		
										TN rate		
			2000 images 1000 benign 1000 malignant							FN rate		
Vaes et al*.* [49]	Prospective Case–control	197	291 adnexal masses	OVHS + RMI1	N/A	N/A	N/A	88%	95%	N/A	Training 70%	N/A
			125 benign	OVHS + RMI2							Testing 30%	
			166 malignant	**OVHS + RMI3**							100 times a random subsampling process	
Vaes et al*.* [50]	Prospective Case–control	197	197 ultrasound images—365 ovarian tumors	LR (1)	(1) 9	N/A	(1) 0.97	(1) 83%	(1) 98%	N/A	Training 60%	RMI (3)
			77—normal	NN (2)	(2) N/A		(2) 0.93	(2) 80%	(2) 86%		Testing 40%	LR2 (4)
			125—benign		(3) 7		(3) 0.80	(3) 69%	(3) 79%		100 bootstrap resampled data sets with AICC selection	NN2 (5)
			166—malignant		(4) 6		(4) 0.85	(4) 79%	(4) 70%			
					(5) 7		(5) 0.87	(5) > 99%	(5) 10%			
Lucidarme et al*.* [52]	Prospective Case–control	264	375 ovaries	OVHS	N/A	N/A	N/A	98%	88%	PPV	One group	N/A
			107 normal							NPV		
			127 benign							TP rate		
			141 malignant							FP rate		
										TN rate		
			359 sonographist opinion							FN rate		
			104 normal ovaries									
			119 benign									
			136 malignant									
Lu et al*.* [51]	N/A	425	425	SVM (1)	(1) 10	(1) 84.38%	(1) 0.918	(1) 85.19%	(1) 83.96%	PPV	Training 62%	RMI (2)
	Case control		291 benign		(2) 7	(2) 76.88&	(2) 0.873	(2) 81.48%	(2) 74.53%	NPV	Testing 38%	LR1 (3)
			134 malignant		(3) 12	(3) 80.63%	(3) 0.911	(3) 81.48%	(3) 80.19%		1 internal test set	LR2 (4)
					(4) 6	(4) 78.75%	(4) 0.916	(4) 81.48%	(4) 77.36%		1 external validation set	
											30-fold cross validation	
Zimmer et al*.* [53]	Retrospective Case–control	163	163 images	Bayes method	4	82.10%		80%	100%	PPV	Training 85%	N/A
			25 transparent cyst							NPV	External validation 15%	
			67 turbid cyst									
			50 significantly solid									
			21 solid									
*b: CAD CT (3)*												
Li et al*.* [54]	Retrospective Case–control	140	140	Radiomics segmentation models	**(1) 10**	(1) 97.6%	(1) 0.99	(1) 95.7%	(1) 100%	N/A	Training 61%	N/A
			62 benign		**4 clinical**	(2) 90.2%	(2) 0.97	(2) 100%	(2) 82.6%		Testing 29%	
			72 malignant		(2) 11						07:03	
					5 clinical							
Park et al. [55]	Retrospective Case–control	427	427	**RF**	8	N/A	0.88	91%	69%	N/A	tenfold cross validation	N/A
			348 benign	LR								
			79 malignant									
Li et al*.* [56]	Retrospective Case–control	160	160 images	Nomogram (int. val)	14	(1) 89.7%	(1) 0.897	(1) 94.7%	(1) 85.0%	N/A	Training 59%	N/A
			134	Nomogram (ext. val)							Testing 24%	
			62 benign			(2) 88.0%	(2) 0.880	(2) 84.6%	(2) 91.7%		External validation 17%	
			72 malignant								tenfold cross validation	
			External dataset N/A									
*c: CAD MRI (6)*												
Liu et al*.* [57]	Retrospective Case–control	196	196	Radiomics segmentation	(1) 396	(1) 99,0%	(1) 1.0	(1) 100%	(1) 98.0%	PPV	Random Training 50% Testing 50%	N/A
			91 borderline	models*						NPV		
			10 malignant	3D sagit (1)	(2) 396	(2) 78.9%	(2) 0.82	(2) 72.9%	(2) 85.1%			
				2D coron (2)								
Song et al*.* [58]	Prospective Case–control	82	104	PK-model	(1) 7	(1) 84.2%	N/A	(1) 66.7%	(1) 100%	N/A	Training 70% Validation 30% 3-class classification task	Radiologists (2)
			33 benign		(2) N/A	(2) 68.4%		66.70%	93.80%		50-fold cross-validation	benign
			18 borderline					70%	77.80%			borderline
			53 malignant					(2) 66.7%	(2) 92.3%			malignant
								66.70%	81.3%%			
								70%	77.80%			
Jian et al*.* ** [59]	Retrospective Case–control	501	501	MICNN	512	76.70%	0.884	74.80%	80.80%	F1 score	Training 68%	N/A
			165 borderline	EMP							(centers A-B)	
			336 malignant	LMP							External validation set 32%	
											(centers C-H)	
Jian et al*.* *** [62]	Retrospective Case–control	501	22,977	MICNN MAC-net	512	82.70%	0.878	N/A	N/A	F1 score	Training 76% Validation 23%	N/A
			501									
			165 borderline									
			336 malignant									
Li et al*.* [61]	Retrospective Case–control	501	501	MP-ST (1)	(1) 851	(1) N/A	(1) 0.920	(1) N/A	(1) N/A	N/A	Training 50%	Radiologists (3)
			165 borderline	CE-T1W1 (2)	(2) 851	(2) N/A	(2) 0.801	(2) N/A	(2) N/A		Internal validation 18%	
			336 malignant		(3) N/A	(3) N/A	(3) 0.797	(3) 80.5%	(3) 78.9%		(centers A-B)	
											External validation 32% (centers C-H)	
Zhang et al*.* [60]	Retrospective Case–control	280	72 benign	SVM (b-m) (1)	(1) 84	(1) 90.6%	(1) 0.9670	(1) 90.3%	(1) 91.3%	PPV	Randomly	Radiologists (3)
			100 type I EOC	SVM (I-II) (2)	(2) 56	(2) 83.3%	(2) 0.8228	(2) 76.5%	(2) 86.5%	NPV	LOOCV 70%	
			81 type 2 EOC		(3) N/A	(3) 83.5%	(3) N/A	(3) 82.3%	(3) 86.9%	TP rate	Testing 30%	
										FP rate		
										TN rate		
										FN rate		

AUC = Area Under the Curve; PPV = positive predictive value; NPV = negative predictive value;SVM = standard vector machine; DCNN = (deep) Convolutional Neural Network); N/A = not applicable; DSS = decision; support system, based on 3 radiomics models VGGNet, ResNet, MobileNet; SA = subjective assessment of an expert (gynaecologist/sonographist); SR = IOTA Simple Rules model; SRL = IOTA simple rules risk model; IDI = integrated discrimination improvement; KNN = k-nearest neighbor; LD = Linear Discriminant; ELM = Extreme Machine Learning (***linear-gaussian in this example); LOO-CV = Leave-One-Out Cross Validation procedure; FCNN = Fully Connected Convolutional Neural Network; RF = Random Forest; FRNN = Fuzzy-Rough Nearest Neighbor; FF = fuzzy forest; MLP Multilayer Perceptron Networks; LBP = Local Binary Pattern; PNN = Probabilistic Neural Network; DT = Decision Tree; ABTA = automatic texture based algorithm; RBF = Radial Basis Function; OVHS = Ovarian HIstoscanning; RMI = Risk of Malignancy; LR = Logistic Regression; NN = Neural Network; AICC = Akaike information corrected criterion; **Bold = best performing classifier**

N/A = not applicable; LR = logistic regression; RF = random forest; **Bold = best performing classifier**

MICNN = Multiple instance convolutional neural network; EMP = early multiparametric; LMP = late multiparametric; PK model = pharmacokinetic model; MP-solid = multiparametric solid tumor model; CE-T1W1 = Contrast-enhanced T1W1 model; **Bold = best performing**

* = radiologist, gynaecologist, sonographist or other(s)

** = Unable to split data set in 70% and 30% training and validation sets, due to limited number malignant tumors, therefore classifier was run twice with different variables

*** = Acharya et al*.* [44—GyneScan: An improved online paradigm for screening of ovarian cancer via tissue characterization

**** = Acharya et al*.*[42]—Evolutionary algorithm-based classifier parameter tuning for automatic ovarian cancer tissue characterization and classification

* Different segmentation models were constructed using 3D and 2D MRI in coronal and sagittal plane;

**Jian et al*.* [59]—MRI-Based Multiple Instance Convolutional Neural Network for Increased Accuracy in the Differentiation of Borderline and Malignant Epithelial Ovarian Tumours;

***Jian et al*.* [64]—Multiple instance convolutional neural network with modality-based attention and contextual multi-instance learning pooling layer for effective differentiation between borderline and malignant epithelial ovarian tumours

In the included studies, fourteen different machine learning modalities were employed: Seventeen were different types of deep machine learning, and the remaining were conventional machine learning. Fourteen studies used classification [34, 39–47, 50, 51, 53, 63], and remaining studies used segmentation to predict the nature of the ovarian tumor. With classification, a class label (e.g., benign or malignant) is predicted by analyzing its input, which is often numerical data (e.g., images). With segmentation, each pixel in an image is assigned to a predefined category (e.g., malignant or non-malignant), whereby certain image characteristics are shared by pixels with identical labels [64]. The input for the segmentation studies was usually different types of grayscale patterns, e.g., gray-level size zone matrix or wavelet features. The input for the classification studies was global images with or without clinical variables added.

### Pooling of diagnostic accuracy

A meta-analysis on the seven studies that used an external validation set to test their CAD model was attempted; however, due to heterogeneity, missing diagnostic accuracy rates and unclear data, this could not be executed [33, 35, 51, 53, 56, 59, 61]. An additional sub-analysis of studies using CAD on ultrasound imaging was performed, which showed great heterogeneity as well. The remaining twenty-four studies without an independent validation per imaging modality were not pooled due to heterogeneity at forehand.

### Risk of bias in studies per imaging modality

A general overview of risk of bias of per imaging modality of the included studies is presented in the ‘Risk of bias’ summary (Table 2a–c).

**Table 2:** 'Risk of bias' summary: review authors' judgements about each risk of bias item for each included study

	Participants	Predictors	Outcome	Analysis	CAD model
*'Risk of bias' summary: Per item for each included study—Ultrasound*					
Gao et al*.* [33]	**Low**	**Low**	**Low**	High	**Low**
Chiappa et al*.* [34]	**Low**	**Low**	**Low**	*Unclear*	*Unclear*
Chiappa et al*.* [35]	**Low**	**Low**	**Low**	**Low**	*Unclear*
Christiansen et al*.* [36]	**Low**	**Low**	**Low**	**Low**	**Low**
Qi et al*.* [37]	**Low**	**Low**	**Low**	**Low**	High
Stefan et al*.* [63]	*Unclear*	**Low**	**Low**	**Low**	High
Wang et al. [38]	**Low**	**Low**	**Low**	*Unclear*	*Unclear*
Martinez-Mas et al. [39]	High	**Low**	**Low**	High	High
Zhang et al. [40]	High	*Unclear*	High	High	**Low**
Acharya et al. [41]	High	**Low**	**Low**	High	High
Aramendia-Vidaurreta et al. [46]	*Unclear*	**Low**	**Low**	**Low**	*Unclear*
Khazendar et al. [47]	*Unclear*	**Low**	**Low**	**Low**	High
Acharya et al. [44]	High	**Low**	**Low**	High	High
Acharya et al. [42]	High	**Low**	High	High	High
Acharya et al. [45]	*Unclear*	*Unclear*	**Low**	High	High
Faschingbauer et al. [48]	**Low**	**Low**	**Low**	High	*Unclear*
Acharya et al. [43]	High	**Low**	High	High	*Unclear*
Vaes et al. [49]	*Unclear*	**Low**	**Low**	High	*Unclear*
Vaes et al. [50]	**Low**	**Low**	**Low**	**Low**	*Unclear*
Lucidarme et al. [52]	**Low**	**Low**	**Low**	*Unclear*	High
Lu et al. [51]	**Low**	**Low**	**Low**	**Low**	**Low**
Zimmer et al. [53]	High	**Low**	*Unclear*	High	*Unclear*
*'Risk of bias' summary: Per item for each included study—CT*					
Li et al*.* [54]	**Low**	**Low**	**Low**	**Low**	**Low**
Park et al*.* [55]	**Low**	**Low**	High	**Low**	**Low**
Li et al*.* [56]	**Low**	**Low**	**Low**	**Low**	**Low**
*'Risk of bias' summary: Per item for each included study—MRI*					
Liu et al*.* [57]	**Low**	**Low**	**Low**	**Low**	*Unclear*
Song et al*.* [58]	**Low**	**Low**	**Low**	High	*Unclear*
Jian et al*.* [59]	**Low**	**Low**	**Low**	**Low**	**Low**
Jian et al*.* [62]	**Low**	High	**Low**	*Unclear*	*Unclear*
Li et al*.* [61]	**Low**	**Low**	**Low**	High	**Low**
Zhang et al*.* [60]	**Low**	**Low**	**Low**	**Low**	**Low**

### Ultrasound:

#### Participants

Risk of bias based on selection of participants was considered low in ten studies. In five studies, risk of selection bias was unclear, because inclusion of participants was not clearly described. Seven studies were graded with high risk of selection bias because they described neither little information about baseline patient characteristics nor inclusion or exclusion criteria.

#### Predictors

Risk of bias based on predictors was considered low for twenty studies, because predictors were defined and assessed in the same way for all participants and predictor assessments were made before results were known. For two studies, this was unclear due to missing information on this matter.

#### Outcome

Risk of bias based on outcome or its determination was considered low for eightteen studies, because in these studies the outcome was predetermined appropriately. Risk of bias was scored unclear in in one study, because there was no clear description of the reference standards used and high in three studies, since reference standards were not described.

#### Analysis

Risk of bias based on analysis was considered low for eight studies, because analysis was properly performed. In three studies, risk of bias based on analysis was unclear, because analysis was not clearly described. Eleven studies described very little of the analysis process, and therefore, these studies were considered containing high risk of bias.

#### CAD model

Risk of bias based on CAD model bias screening questions was considered low in four studies. In nine studies, the risk of bias based on CAD model bias screening questions was assessed as unclear, because it was unclear how overfitting mitigation techniques and cross-validation were used or if the data were reproducible or validated in other centers. Risk of bias based on CAD model bias screening questions was reckoned high in nine studies. This was due to overfitting mitigation techniques which were not used or incorrectly used, the training set was not independent from the test set or did not have enough power, or no cross-validation was used and data were not reproducible or not validated in other settings.

### CT and MRI

#### Participants

Risk of bias based on selection of participants was considered low in all nine studies, because of transparent description of patient selection.

#### Predictors

Risk of bias based on predictors was regarded low for eight studies, because the researchers were clear on how predictors were determined and characterized before outcome was known. Only one study reported that the outcome was known at forehand when assessing the predictors.

#### Outcome

Risk of bias based on outcome or its determination was considered low for eight studies, because it was predetermined appropriately. For one study, this was high because outcome differed among participants.

#### Analysis

Risk of bias based on analysis was considered low for six studies, because analysis was accurately carried out. In two studies, risk of bias based on analysis was high, because analysis was not properly performed. In one study, the sub-results were inconclusive and therefore the study was considered containing an unclear risk of bias.

#### CAD model

Risk of bias based on CAD model bias screening questions was assessed low in six studies. In three studies, risk of bias based on CAD model bias screening questions was considered unclear, because the use of overfitting mitigation techniques was not mentioned or they were not executed correctly, and it was unclear if executed correctly it was unclear if the dataset was reproducible or validated in other settings.

## Discussion

This systematic review shows numerous studies that use CAD to assess the nature of an ovarian tumor. Due to the large heterogeneity, we were not able to pool data. However, highest performance as measured by AUC was seen in both CT- and MRI-based CAD models.

A meta-analysis was endeavored for the seven studies that used an external dataset for validation. However, this could not be executed for multiple reasons. One study, describing a CAD-MRI model for differentiating borderline from malignant ovarian tumors, only mentioned the sensitivity and specificity for radiologists’ performance and for the model only the AUC [61]. Another study was unclear about which data were used to calculate the diagnostic performance of their model [56]. Consequently, for both studies it was not possible to calculate diagnostic accuracy rates, such as true positive (TP), true negative (NT) values and to use them in the meta-analysis.

For the five remaining studies, heterogeneity proved to be too large with an I2 of 92.8% and 90.7%. In an additional subgroup analysis of only ultrasound CAD models, this was also apparent with an I2 of 94.3% and 83.5%. These analyses can be found in Additional file 1: Appendix 3. This heterogeneity can be explained by (1) different types of CAD models using either conventional or deep learning techniques, (2) different inclusion and exclusion criteria and (3) type of imaging modality used. Among the twenty-four studies without an independent dataset, pooling of the results was not viable since the data were too diverse. This was illustrated by differences in imaging techniques used, e.g., 2D or 3D ultrasound and CT, or 2D, 3D or pharmacokinetic MRI. Furthermore, different CAD techniques were applied, e.g., conventional and deep learning machine learning models. Moreover, some studies combined clinical features such as patients’ age, menopausal status or serum CA125 to support the classifiers. Finally, different outcome measurements per classifier were found, such as benign, malignant and borderline in combination with a different tumor subtype, such as mucinous ovarian tumors.

All studies assessed computer-aided diagnostics based on machine learning. We found that classifying the nature of an ovarian tumor by CAD on ultrasound images results in sensitivities of 40.3% to 100% and specificities of 76.3% to 100%. For CT, sensitivities of 84.6% to 100% and specificities of 69% to 100% were described. For MRI, sensitivities and specificities ranged between 66.7% and 100% and 77.8% and 100%, respectively. Even though some studies report high performances, they are at risk for overfitting due to the lack of an independent test set. Twenty-three studies lacked an independent test set for evaluating model performance.

With conventional machine learning techniques, features extracted from medical imagery are used to optimize a mathematical model for predicting new, unseen data. A model should be built based on a training set of images and validated in a test set. If the model is too tightly fitted to the training data and does not generalize toward new data, it is called overfitting. Overfitting occurs more often with conventional machine learning, where many parameters are hand-selected instead of being learned from the data, especially when the model is not validated on an independent test set [64].

### Ultrasound

Earlier published studies assessing ultrasound prediction models show reasonable sensitivity (72–77%) and specificity (85–89%) for the RMI [65, 66]. An external validation of the IOTA ADNEX model showed a better performance, with a sensitivity of 98% (95% CI 93–100%), but with low specificity of 62% at a cutoff value for malignancy of 10% (95% CI 55%–68%) [5]. The GI-RADs and the O-RADs perform better with a sensitivity of 92.7% and 93.6% and a specificity of 97.5% and 92.8%, subsequently [6]. However, all these models depend on specific terminology and expertise of their users. Furthermore, interpretation of ultrasound imaging regarding ovarian tumors has shown to be difficult for novel clinicians and for clinicians who do not perform ultrasonography on a regular basis [8, 9]. Based on the amount of studies included in this review assessing the CAD technique for ultrasound, CAD can be a promising tool to aid clinicians in determining the origin of ovarian tumors. Moreover, when comparing CAD models’ performances with experienced clinicians or existing models they achieve similar or even better diagnostic accuracy. Nevertheless, this performance comparison was performed in only three studies. Even though overfitting mitigation techniques were applied in twenty-one ultrasound studies, only four studies used external validation. Thus, a high risk of overfitting is present, which could lead to an unreliable performance.

### CT

The diagnostic performance of CT in preoperatively classifying the origin of an ovarian tumor is primary known for multidetector computer tomography (MDCT), with a diagnostic accuracy of 90–93% [11]. Therefore, no fair comparison on CAD for CT can be made. However, the performance of CAD for CT is indeed promising based on the included studies in this review. The models show a high diagnostic accuracy and low selection bias. Nonetheless, only three studies in total assessed CAD for CT of which only one study utilized an independent validation, thus risking overfitting.

For CAD on CT scans, more research is needed to further evaluate its potential benefits.

### MRI

The diagnostic accuracy for MRI in ovarian tumor classification has a sensitivity and specificity of 96% and 91%, respectively [7, 9]. For the O-RADs MRI score, this is comparable with a sensitivity of 93% and a specificity of 91% and it shows a similar performance among junior and senior radiologists (κ = 0.784; 95% CI, 0.743–0824) [9, 10]. CAD for MRI as an additional diagnostic method for ovarian tumors has the potential to aid radiologists due to its high diagnostic performance as a single model or when compared to SA of radiologists. However, caution is needed when using MRI-CAD as a supplementary tool. First, due to the absence of international guidelines when to conduct an MRI for ovarian tumors classification a selection bias is being created. Moreover, the performance of the MRI has no further clinical consequences for the patient. However, if radiologists are trained with MRI O-RADs classification model, the usage of MRI can have an additional beneficial effect on ovarian tumor classification, especially when classifying benign and or possibly malignant lesions [67]. However, for the O-RADs MRI familiarity and expertise are essential to use the scoring system [7, 10].

Second, only one out of six studies showed a low overall risk of bias on using MRI CAD [59]. Unfortunately, the authors did not compare their CAD to ovarian tumor characterization by radiologists or to other models, such as the O-RADs model. Hence, one study alone cannot support clinical implementation of MRI CAD. Moreover, although in three studies CAD outperformed the radiologists’ performance, no external validation sets were used in these studies and risk of bias was mostly unclear [58, 59, 60, 61]. Furthermore, only two of the six studies used an external validation set [59, 61]. Another study used 3D MRI for their model building, showing good results; however, this is a rather expensive MRI technique [57]. Finally, two studies used the same dataset. Therefore, only limited evidence to support the usage of MRI-CAD additionally is available [59, 63].

Hence, more studies should be undertaken with external validation sets in order to be able to implement these CAD-MRI models in clinical practice.

### Trends among publications

Over the last three decades, different trends among included studies in the CAD field are observed.

An increasing number of publications presented clear inclusion and exclusion criteria for data before using it to construct a CAD model [33–39, 44, 48–50, 52, 54–63]. In addition, more studies used statistical tests to select the most promising features to include into the CAD model and in most articles this was precisely described [34, 37, 41, 43–46, 48–51, 54–58, 60–63]. Furthermore, study cohorts became substantially larger [33]. Finally, clinicians are more involved in the CAD model construction, e.g., for the delineation of the images. Thus, uniformity among studies is improved, making studies more comparable.

Regarding the outcomes, almost all studies used the same outcome measurements, i.e., sensitivity, specificity, accuracy and area under the curve (AUC). More connection with the clinical setting is observed. In particular, the comparison of the CAD model to either assessment of scans by clinicians such as radiologists, sonographists or gynecologists or to commonly used models in ultrasound (RMI or LR1-2) is now included [33, 35–38, 48, 51, 60, 61, 68].

Hence, the difficult﻿ technical matter of a CAD model development is made more comprehensible for clinicians.

Finally, more deep learning models have been developed in recent years, showing the potential of this new type of CAD. If these trends continue, it could substantially contribute to patient care.

Previous studies have shown that depending on the imaging technique used the interobserver agreement is low for many features and are prone to contain significant measurement errors when used by inexperienced clinicians. Therefore, more uncertainties in measured features within these imaging techniques can lead to diminished accuracies of a model. It is therefore important to develop new techniques with less inter- and intra-observer variability to reach higher test performances to prevent unnecessary referrals to tertiary centers and unnecessary stress for the patient. Based on this literature review, computer-aided ultrasound, CT and MRI techniques based on different (deep) neural networks and conventional machine learning techniques such as support vector machines are promising. They can either be used as a single entity or combined with SA or with other prediction models. They could potentially offer a noninvasive and cost-effective method in the future. However, this is only shown in eight studies of which five are ultrasound studies and three MRI studies. Of these studies, four used independent validation sets, of which three within ultrasound CAD and one within an MRI CAD. For the remaining studies, lack of a validation cohort might cause a high risk of overfitting. The CT CAD models seem to perform fairly but they consist of small datasets and are in the absence of a SA and only one study used an external validation set; therefore, risk of overfitting is present.

Furthermore, CAD as a technique within the gynecology–oncology is slowly gaining field in comparison with other oncology specialties. Combining datasets with larger test sets is needed in prospective cohorts [22, 33, 69].

It is likely that deep learning in assessing the nature of an ovarian tumor will reach higher test performances than traditional machine learning. For MRI and CT, the number of studies in this review is limited and needs to be broadened [22].

### Strengths and weaknesses

To the best of our knowledge, this is the most comprehensive review on computer-aided diagnostics for differentiating benign from borderline and malignant ovarian tumors on ultrasound, MRI and CT scans. We have worked by a clearly defined protocol that was first submitted to PROSPERO, to provide transparency in the review process and avoid reporting bias. There was no substantial disagreement in inclusion of articles by the authors, and this can be regarded as a strong point in the review process. A meta-analysis of the studies with an external validation set was attempted. A limitation of this review is the heterogeneity between studies, the lack of independent validation sets and comparison with SA.

## Conclusions

In conclusion, this review shows that CAD certainly has potential as a noninvasive model to preoperatively predict whether an ovarian tumor is benign, borderline or malignant and thus can aid the physician with assessment of ovarian tumors. However, this depends on the type of imaging modality assessed and thus should be evaluated per imaging technique. CAD for CT displays the best performance overall. However, the three studies included are all lacking an external validation. The results of CAD for MRI were similar; however, more studies used external validation to test their CAD. Nevertheless, the risk of bias for the domain ‘CAD model’ for half of the studies was found to be unclear. Furthermore, it is important to take into account that MRI is clinically less relevant for detecting and classifying ovarian tumors. Finally, most research has been done on CAD for ultrasound, of which the results are reasonable in comparison with existing models, but has limited external validation and risks overfitting. Moreover, included studies per image modality show great heterogeneity, and thus, results most likely cannot be generalized to other data.

Studies in which all methods are validated in the same population should be performed in order to prove which techniques demonstrate the best diagnostic performance. Above all, it is important that new CAD techniques are tested and validated with an independent, prospectively collected dataset.

### Future perspectives

In the near future, it is likely that CAD will facilitate diagnostics and will be used as a decision support system by clinicians, depending on the imaging modality the CAD is developed for. The performance of CAD for discriminating the nature of an ovarian tumor on CT and MRI is good, and studies assessing these two imaging techniques show a low risk of bias. Consequently, a majority of research should focus on these two imaging modalities. Particularly, since both MRI and CT are more standardized than ultrasound imaging and therefore more suitable for CAD development. However, it should be taken into account that MRI is less clinically relevant in diagnosing ovarian tumors. In addition, in order to increase accuracy, CAD for CT or MRI could be combined with clinical markers, e.g., menopausal age or liquid biopsies, such as circulating cell free tumor DNA (ct-DNA). Implementation of CAD for ultrasound in clinical practice will presumably take longer due to the dynamic character of this imaging method and the high and unclear risk of bias.

## Supplementary Information

**Additional file 1**. Appendix 1. Search syntax. Appendix 2. Signaling questions. Appendix 3. Meta-analysis results.

**Additional file 2**. Tables 1a–c.

## Data Availability

Data sharing is not applicable to this article as no datasets were generated or analyzed during the current study.

## Abbreviations

ADNEX model: Assessment of different NEoplasias in the adneXa
CA125: Cancer antigen 125
CAD: Computer-aided diagnostics
CT: Computer tomography
ct-DNA: Circulating cell-free tumor DNA
GI-RADs: Gynecologic imaging reporting and data system for diagnosis of adnexal masses (AMs) by pelvic ultrasound (US)
MDCT: Multidetector computer tomography
MRI: Magnetic resonance imaging
O-RADs: Ovarian-adnexal reporting and data system
PROBAST: Prediction Model Study Risk of Bias Assessment Tool
QUADAS-2: Quality Assessment of Diagnosis Accuracy Study
QUIPS: Quality in prognostic studies
RMI: Risk of malignancy index
ROI: Region of interest
SA: Subjective assessment
SROC: Summary receiver operating curve
TP: True positive
TN: True negative
