# Supplementary material for: Analysis of computer-aided diagnostics in the preoperative diagnosis of ovarian cancer: a systematic review
Source: Insights Imaging. 2023 Feb 15;14:34. doi: 10.1186/s13244-022-01345-x (PMC9931983; doi:10.1186/s13244-022-01345-x)

Appendix 1: Search syntax per database

Pubmed search

(Ovarian neoplasm*[tiab] OR "Ovarian Neoplasms"[Mesh] OR "ovarian mass" [tiab] OR "ovarian masses" [tiab] OR "ovarian lesion*" [tiab] OR "ovarian tumor*" [tiab] OR ''ovaria'' [tiab] OR ''adnexal''[tiab] OR ''adnexal mass''[tiab] OR ''ovarian cancer''[tiab] OR ''ovarian malignancy'' [tiab] OR ovary [Mesh] OR ovarian mass*[MeSH] OR classification of ovarian [tiab] OR classification of ovarian [Mesh])

AND

("neural network" [tiab] OR ''machine learning''[tiab] OR "Machine Learning" [Mesh] OR "artificial intelligence" [tiab] OR "Artificial Intelligence"[Mesh] OR "decision support technique" [tiab] OR "decision support techniques" [tiab] OR "Decision Support Techniques"[Mesh] OR "machine learning classifier" [tiab] OR "machine learning classifiers" [tiab] OR "computer assisted" [tiab] OR "computer aided" [tiab] OR "Diagnosis, Computer-Assisted"[Mesh] OR "computer-assisted diagnosis" [tiab] OR ''radiomics'' [tiab] OR "Neural Networks, Computer" [Mesh] OR convolutional neural network [tiab] or convolutional neural network [Mesh])

AND

("diagnostic accuracy" [tiab] OR "diagnosis" [tiab] OR "Presurgical" [tiab] OR "Preoperative" [tiab] OR preoperative diagnosis [tiab] OR preoperative diagnosis [Mesh])

AND

("ct-scan" [tiab] OR ''ultrasound'' [tiab] OR "MRI" [tiab] OR "Tomography, X-Ray Computed"[Mesh] OR "magnetic resonance imaging" [tiab] OR "Magnetic Resonance Imaging"[Mesh] OR "gynaecological ultrasound" [tiab] OR "Ultrasonography"[Mesh] OR "Ultrasonography" [tiab])

Embase search

(Ovarian neoplasm.kw,ti,ab. OR ovarian mass.kw,ti,ab. OR ovarian masses.kw,ti,ab. OR ovarian lesion.kw,ti,ab. OR ovarian tumor.kw,ti,ab. OR ovaria.kw,ti,ab. OR adnexal.kw,ti,ab. OR adnexal mass.kw,ti,ab. OR ovarian malignancy.kw,ti,ab. OR ovary tumor/ OR ovary/ OR ovarian mass* OR classification of ovarian.kw,ti,ab. OR ovary carcinoma/ OR ovary cancer/)

AND

(neural network.kw,ti,ab. OR machine learning.kw,ti,ab. OR artificial intelligence.kw,ti,ab. OR decision support technique.kw,ti,ab. OR decision support techniques.kw,ti,ab. OR machine learning classifier.kw,ti,ab. OR machine learning classifiers.kw,ti,ab. OR computer assisted.kw,ti,ab. OR computer aided.kw,ti,ab. OR computer assisted diagnosis.kw,ti,ab. OR radiomics.kw,ti,ab. OR convolutional neural network.kw,ti,ab. OR artificial neural network/ OR convolutional neural network/ OR machine learning/ OR artificial intelligence/ OR decision support system/ OR computer assisted diagnosis/ OR radiomics/)

AND

(diagnostic accuracy.kw,ti,ab. OR diagnosis.kw,ti,ab. OR presurgical.kw,ti,ab. OR preoperative.kw,ti,ab. OR preoperative diagnosis.kw,ti,ab OR preoperative evaluation/)

AND

(CT scan.kw,ti,ab. OR ultrasound.kw,ti,ab. OR magnetic resonance imaging.kw,ti,ab. OR ultrasonography.kw,ti,ab. OR gynaecological ultrasound.kw,ti,ab. OR x-ray computed tomography/ OR ultrasound/ OR nuclear magnetic resonance imaging/ OR echography/)

Medline search

(Ovarian neoplasm$.mp. or Ovarian Neoplasms/ or ovarian mass.mp. or ovarian masses.mp. or ovarian lesion$.mp. or ovarian tumor$.mp. or ovaria.mp. or adnexal.mp. or adnexal mass.mp. or ovarian cancer.mp. or ovarian malignancy.mp. or ovary/ OR ovarian mass* OR classification of ovarian.mp)

AND

((neural network or machine learning).mp. or Machine Learning/ or artificial intelligence.mp. or Artificial Intelligence/ or decision support technique$.mp. or Decision Support Techniques/ or machine learning classifier.mp. or machine learning classifiers.mp. or computer assisted.mp. or computer aided.mp. or Diagnosis, Computer-Assisted/ or computer-assisted diagnosis.mp. or radiomics.mp. or Neural Networks, Computer/ OR convolutional neural network.mp)

AND

((diagnostic accuracy or diagnosis or presurgical or preoperative or preoperative diagnosis).mp.)

AND

((ct-scan or ultrasound or MRI).mp. or Tomography, X-Ray Computed/ or magnetic resonance imaging.mp. or Magnetic Resonance Imaging/ or gynaecological ultrasound.mp. or Ultrasonography/ or Ultrasonography.mp.)

Scopus search

TITLE-ABS-KEY (ovarian AND neoplasm* OR ovarian AND mass* OR ovarian AND lesion* OR ovarian AND tumor* OR ovaria OR adnexal OR adnexal AND mass* OR ovarian AND cancer* OR ovarian AND malignancy* OR ovary OR classification AND of AND ovarian*) AND TITLE-ABS-KEY (neural AND network* OR machine AND learning* OR artificial AND intelligence* OR decision AND support AND technique* OR machine AND learning AND classifier* OR computer AND assisted OR computer AND aided OR computer-assisted AND diagnosis OR radiomics OR computer AND neural AND networks OR convolutional AND neural AND network) AND TITLE-ABS-KEY (diagnostic AND accuracy OR diagnosis OR presurgical OR preoperative OR preoperative diagnosis) AND TITLE-ABS-KEY (ct-scan* OR ultrasound OR mri OR x-ray AND computed AND tomography OR magnetic AND resonance AND imaging OR gynaecological AND ultrasound OR ultrasonography)

Clinicaltrial.gov

'ovarian tumor', ' ovarian cancer', ' computer-aided diagnostic' ,' machine learning', 'artificial intelligence', ' radiomics'

Cochrane

‘ ovarian cancer’

AND

‘ artificial intelligence’

AND

‘computer tomography (CT)’

Appendix 2: signaling questions to assess risk of bias based on CAD models

1. Were overfitting mitigation techniques used? If described then low risk of bias.
2. Was the training set different from the validation set? If yes then low risk of bias
3. Was cross-validation used? If yes then high risk of bias, because high risk of overfitting when used.
4. Was the dataset made public and reproducible? If yes then low risk of bias
5. Was the model validated in other centers? If yes then low risk of bias
6. Was the test set large enough with enough power? If yes then low risk of bias
7. Were the groups randomly determined? If yes then high risk of bias
8. Were the features analyzed and is analyzed whether the features were significant? If yes then low risk of bias

Appendix 3: Meta-analysis results

Figure 1a: Forest plot of all studies with external validation set

Study	TP	FP	FN	TN	Sensitivity (95% CI)	Specificity (95% CI)
Gao2 2022	96	61	70	662	0.58 [0.50, 0.65]	0.92 [0.89, 0.93]
Gao1 2022	27	4	40	264	0.40 [0.28, 0.53]	0.99 [0.96, 1.00]
Jian 2021	80	10	27	42	0.75 [0.65, 0.83]	0.81 [0.67, 0.90]
Chiappa 2021	20	3	0	12	1.00 [0.83, 1.00]	0.80 [0.52, 0.96]
Lu 2003	114	47	20	244	0.85 [0.78, 0.91]	0.84 [0.79, 0.88]
Zimmer 2003	20	0	5	3	0.80 [0.59, 0.93]	1.00 [0.29, 1.00]
					0.688 [0.646, 0.728]	0.906 [0.889, 0.921]

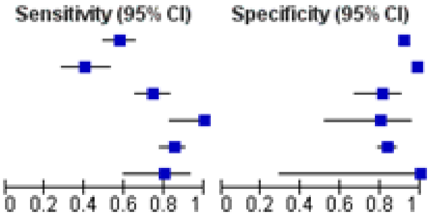

Chi-squared = 69.17 (d.f.= 5), p = 0.000, I² = 92.8 %

Chi-squared = 53.54 (d.f.= 5), p = 0.000, I² = 90.7 %

Table 1a: Summary Sensitivity

Study	Sen	[95% Conf. Iterval.]		TP/(TP+FN)	TN/(TN+FP)
Gao1 2022	0.403	0.285	- 0.530	27/67	264/268
Gao2 2022	0.578	0.499	- 0.654	96/166	662/723
Chiappa 2021	1.000	0.832	- 1.000	20/20	12/15
Jian 2021	0.748	0.654	- 0.827	80/107	42/52
Lu 2003	0.851	0.779	- 0.906	114/134	224/271
Zimmer 2003	0.800	0.593	- 0.932	20/25	3/3
Pooled Sensitivity	0.688	0.646	- 0.728		

Heterogeneity chi-squared = 69.17 (d.f.= 5) | p = 0.000 | Inconsistency (I-square) = 92.8 % | No. studies = 6.

Table 2b: Summary Specificity

Study	Spec	[95% Conf. Iterval.]	TP/(TP+FN)	TN/(TN+FP)
Gao1 2022	0.985	0.962 - 0.996	27/67	264/268
Gao2 2022	0.916	0.893 - 0.935	96/166	662/723
Chiappa 2021	0.800	0.519 - 0.957	20/20	12/15
Jian 2021	0.808	0.675 - 0.904	80/107	42/52
Lu 2003	0.827	0.776 - 0.870	114/134	224/271
Zimmer 2003	1.000	0.292 - 1.000	20/25	3/3
Pooled Specificity	0.906	0.889 - 0.921		

Heterogeneity chi-squared = 53.54 (d.f.= 5) | p = 0.000 | Inconsistency (I-square) = 90.7 % | No. studies = 6.

Figure 1b: SROC-plot of all studies with an external validation set

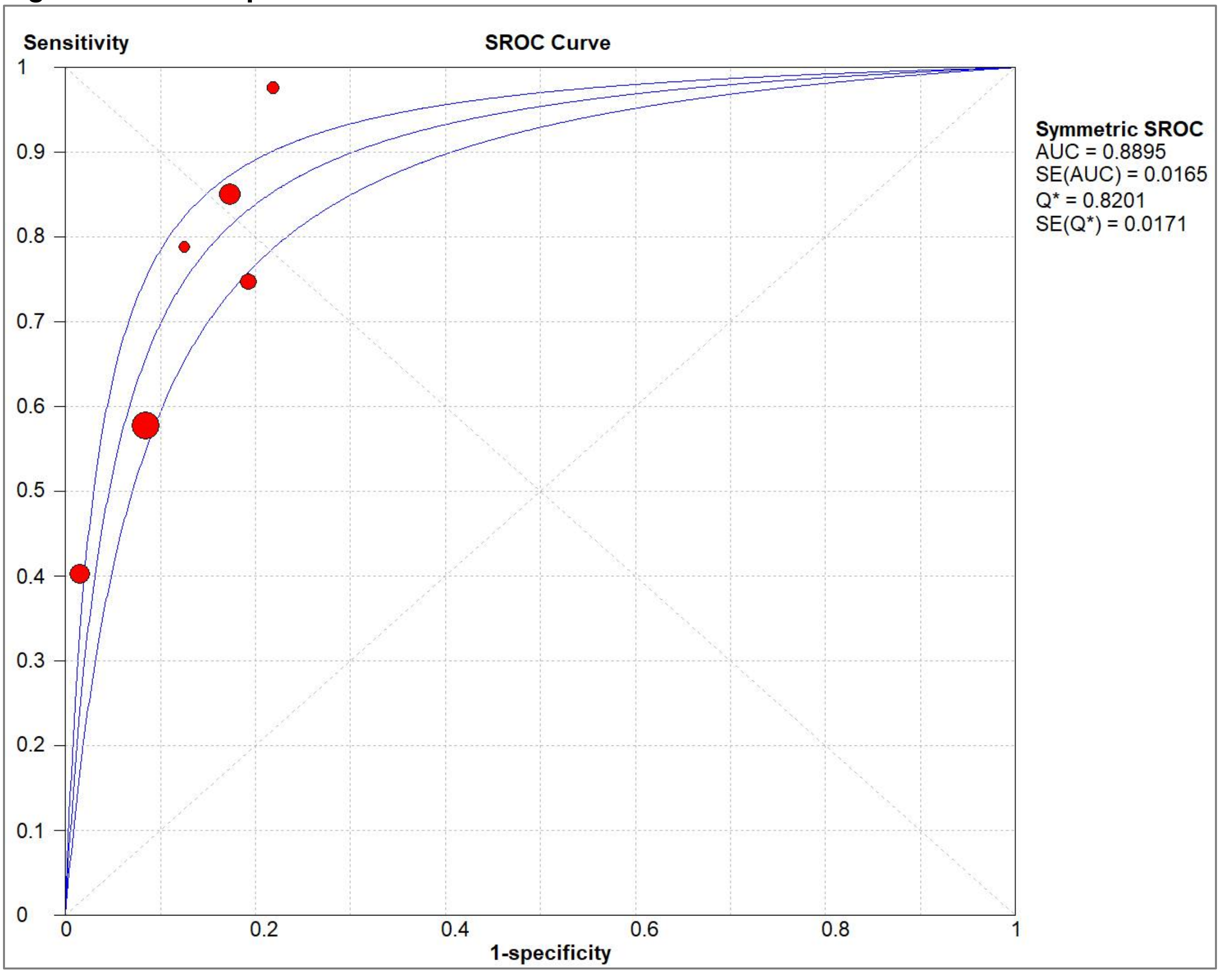

Figure 2a: Forest plot of ultrasound studies with an external validation set

Study	TP	FP	FN	TN	Sensitivity (95% CI)	Specificity (95% CI)
Gao1 2022	20	3	0	12	1.00 [0.83, 1.00]	0.80 [0.52, 0.96]
Gao2 2022	96	61	70	662	0.58 [0.50, 0.65]	0.92 [0.89, 0.93]
Chiappa 2021	27	4	40	264	0.40 [0.28, 0.53]	0.99 [0.96, 1.00]
Zimmer 2003	20	0	5	3	0.80 [0.59, 0.93]	1.00 [0.29, 1.00]
Lu 2003	114	47	20	224	0.85 [0.78, 0.91]	0.83 [0.78, 0.87]
					0.676 [0.627, 0.721]	0.937 [0.925, 0.948]

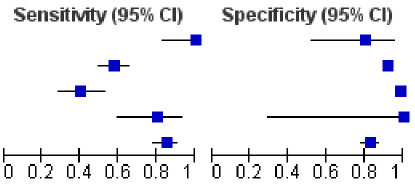

Chi-squared = 69.58 (d.f.= 4), p = 0.000, I^2 = 94.3 %
Chi-squared = 53.54 (d.f.= 4), p = 0.000, I^2 = 83.5 %

Table 2a: Summary Sensitivity

Study	Sen	[95% Conf. Iterval.]	TP/(TP+FN)	TN/(TN+FP)
Gao1 2022	0.403	0.285 - 0.530	27/67	264/268
Gao2 2022	0.578	0.499 - 0.654	96/166	662/723
Chiappa 2021	1.000	0.832 - 1.000	20/20	12/15
Lu 2003	0.851	0.779 - 0.906	114/134	774/821
Zimmer 2003	0.870	0.664 - 0.972	20/23	5/5
Pooled Sen	0.676	0.628 - 0.721		

Heterogeneity chi-squared = 69.58 (d.f.= 4) p = 0.000 | Inconsistency (I-square) = 94.3 % |No. studies = 5.

Table 2b: Summary Specificity

Study	Spec	[95% Conf. Iterval.]	TP/(TP+FN)	TN/(TN+FP)
Gao1 2022	0.985	0.962 - 0.996	27/67	264/268
Gao2 2022	0.916	0.893 - 0.935	96/166	662/723
Chiappa 2021	0.800	0.519 - 0.957	20/20	12/15
Lu 2003	0.943	0.925 - 0.958	114/134	774/821
Zimmer 2003	1.000	0.478 - 1.000	20/23	5/5
Pooled Specificity	0.937	0.925 - 0.948		

Heterogeneity chi-squared = 24.25 (d.f.= 4) p = 0.000 Inconsistency (I-square) = 83.5 % |No. studies = 5.

Figure 2b: SROC-plot of ultrasound studies with an external validation set

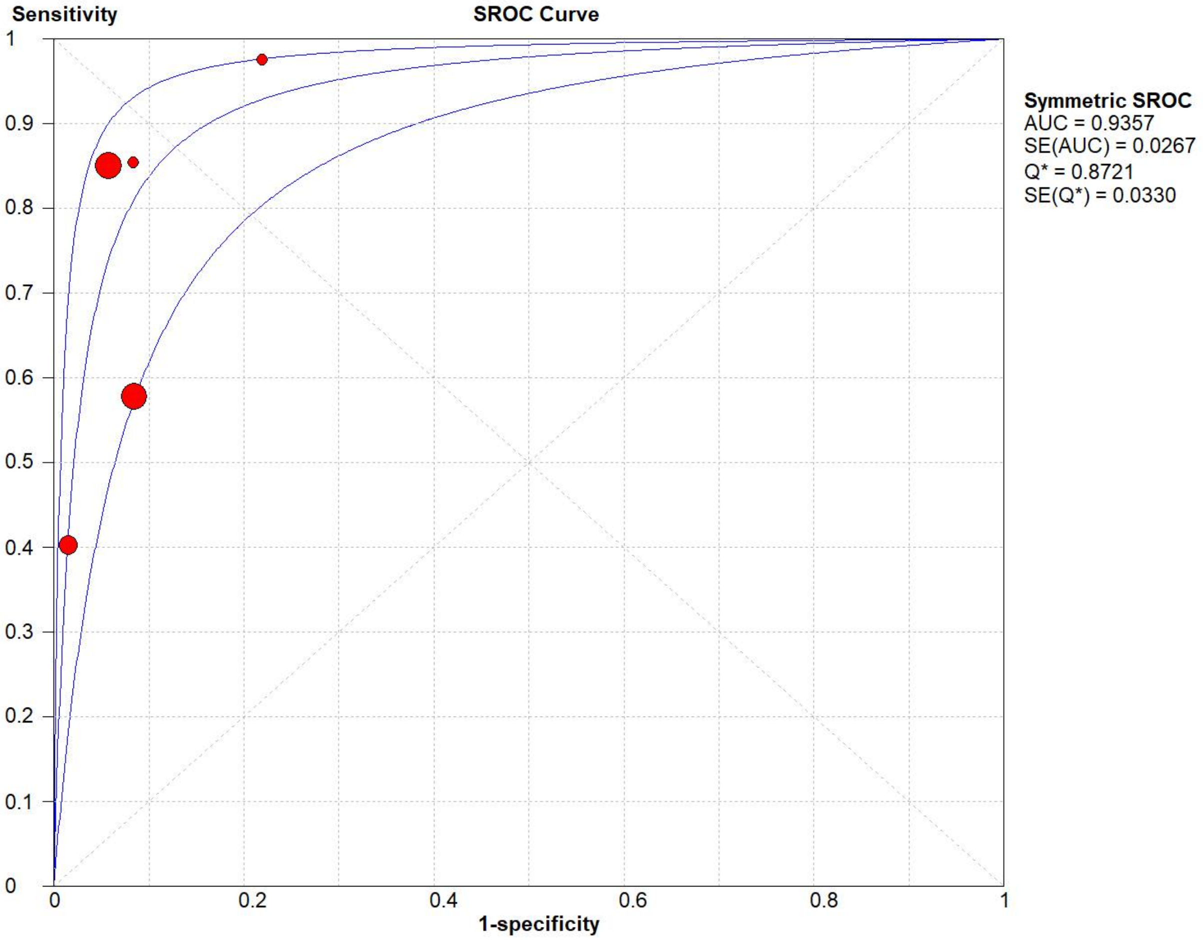

Supplement: Supplementary file 1 — Additional file 1. Appendix 1. Search syntax. Appendix 2. Signaling questions. Appendix 3. Meta-analysis results. [file 13244_2022_1345_MOESM1_ESM.pdf]
