# Supplementary material for: Analysis of computer-aided diagnostics in the preoperative diagnosis of ovarian cancer: a systematic review
Source: Insights Imaging. 2023 Feb 15;14:34. doi: 10.1186/s13244-022-01345-x (PMC9931983; doi:10.1186/s13244-022-01345-x)
Supplement: Supplementary file 2 — Additional file 2. Tables 1a–c. [file 13244_2022_1345_MOESM2_ESM.docx]

**Tables**

**Table 1a-c. Characteristics of included studies**

**Table 1a Characteristics of included studies per image modality ultrasound (22)**

| **Gao *et al.* 2022 (#1)^33^** | | | |
| --- | --- | --- | --- |
| **Title** Deep learning-enabled pelvic ultrasound images for accurate diagnosis of ovarian cancer in China: a retrospective, multicentre, diagnostic study | | |  |
| **Objective** (1) Developed a DCNN model that automated detecting adnexal masses in ultrasound images and discriminating between malignant and benign masses and validated the model internally and externally.  (2) Compared the DCNN model with 35 radiologists and explored whether it could augment the diagnostic accuracy of radiologists. | | |  |
| Methods | **Country** | China |  |
|  | **Funding** | National Key Basic Research Program of China, National Sci-Tech Support Projects, and National Natural Science Foundation of China. |  |
|  | **Design** | Retrospective, case-control |  |
| Participants | **Population description** | See inclusion and exclusion criteria |  |
|  | **Inclusion criteria** | (1) (Age (years)d ≥ 18years) who presented with adnexal lesions in ultrasound in ten hospitals. (2) (Age (years)d ≥ 18years) women whose ultrasonography showed no adnexa- related abnormalities |  |
|  | **Exclusion criteria** | (1) Duplicated cases (2) Postoperative patients who were deprived of adnexa (3) Patients without histological diagnosis |  |
|  | **Number** | 107624 - total  105532 - training set 101777  benign 3755 malignant 868  internal validation 602 - benign 266 malignant  335 ext. validation 1 - 268 benign 67 malignant  889 cases ext. validation 2 - 723 benign 166 malignant |  |
| Model | **Imaging technique** | 2D ultrasound |  |
|  | **CAD model** | DenseNet ((d)CNN = (deep) Convolutional Neural Network) |  |
|  | **Features** | - Weighted cross-entropy loss was used as the objective function.  - The network was trained end-to-end with stochastic gradient descent using an initial learning rate of 0∙001, momentum of 0∙9, weight decay of 0∙0001, and mini batches of 32. The learning rate was decreased by 0∙1 after every 20 epochs.  - Data augmentations during the training process included random resize and crop, random horizontal flip, random rotation, random color jittering, and normalization.  - 121 layers in the DCNN 1 internal validation set 2 external validation set |  |
|  | **Test(s) used to select significant features** | N/A |  |
|  | **Evaluation setup** | 1 internal validation set  2 external validation set |  |
|  | **ROI (region of interest) annotated** | Unclear (automatically based op DCNN) |  |
| Results | **Samples (n)** | 575930 ultrasound images 541442 training set  benign 34488 malignant 8416  5385 internal validation  benign 3031 malignant 1419  1419 external validation 1  933 benign 486 malignant  6510 external validation 2  5257 benign 1253 malignant in validation sets 79 (16%) borderline |  |
|  | **Age (years)** | ≤45 years 70742 >45 years 36882 |  |
|  | **CA125 (U/ML)** | - |  |
|  | **Menopausal status** | 70742 premenopausal  36882 postmenopausal |  |
|  | **Timeframe scanning for surgery** | - |  |
|  | **Timeframe data collection** | Sep 2003 - May 2019  Sep 2003 - Mar 2018 |  |
|  | **Outcome** | Accuracy  AUC  Sensitivity  Specificity  Brier score  F1 score  PPV  NPV |  |
|  | **Intervention compared to** | Histology and radiologist |  |

| **Chiappa *et al.* 2021 (#529)^34^** | | | |
| --- | --- | --- | --- |
| **Title** The Adoption of Radiomics and machine learning improves the diagnostic processes of women with Ovarian MAsses (the AROMA pilot study) | | |  |
| **Objective** To develop and evaluate the performance of a radiomic and machine learning model applied to ultrasound images in predicting the risk of malignancy of ovarian masses. | | |  |
| Methods | **Country** | Italy |  |
|  | **Funding** | No |  |
|  | **Design** | Retrospective, case-control |  |
| Participants | **Population description** | See inclusion and exclusion criteria |  |
|  | **Inclusion criteria** | (1) diagnosis of ovarian mass  (2) execution of a preoperative ultrasonographic examination within 2 weeks before surgery  (3) surgery performed. |  |
|  | **Exclusion criteria** | (1) Age (years) <18 years  (2) absence of ultrasonographic images stored  (3) consent withdrawn |  |
|  | **Number** | 241 |  |
| Model | **Imaging technique** | 2D transvaginal, 3D transvaginal |  |
|  | **CAD model** | Standard vector machines (SVM) |  |
|  | **Features** | Radiomic feature extraction with  (1) TRACE4 segmentation tool with slices thickness of 1mm intensity fixed number of 64 bins  (GLCM) = Morphology, intensity-based statistics, intensity histogram, gray-level co-occurrence matrix  (GLRLM) = gray-level run length matrix  (GLSZM) = gray-level size zone matrix  (NGTDM) = neighborhood gray tone difference matrix  (GLDZM) = gray-level distance zone matrix  (NGLDM) = neighboring gray level dependence matrix  319 total per OM  269 solid benign vs malignant  278 cystic benign vs malignant  306 motley benign vs malignant total 853 |  |
|  | **Test(s) used to select significant features** | Test-retest study (ICC) |  |
|  | **Evaluation setup** | training-validation-testing (nested tenfold validation) |  |
|  | **ROI (region of interest) annotated** | Manually Other: operator |  |
| Results | **Samples (n)** | 241 ultrasound images, 115 benign and 126 malignant |  |
|  | **Age (years)** | 18 - 84, mean 55 |  |
|  | **CA125 (U/ML)** | Median benign - malignant  Solid 17 - 274  Cystic 12 - 92  Motley 11 - 40 |  |
|  | **Menopausal status** | 24% premenopausal; 76% postmenopausal |  |
|  | **Timeframe scanning for surgery** | 2 weeks |  |
|  | **Timeframe data collection** | Jan 2017 - Dec 2019 |  |
|  | **Outcome** | Accuracy  AUC  Sensitivity  Specificity |  |
|  | **Intervention compared to** | Histology |  |

| **Chiappa *et al.* 2021 (#527)^35^** | | | |
| --- | --- | --- | --- |
| **Title** A decision support system based on radiomics and machine learning to predict the risk of malignancy of ovarian masses from transvaginal ultrasonography and serum CA-125. | | |  |
| **Objective** To evaluate the performance of a decision support system (DSS) based on radiomics and machine learning in predicting the risk of malignancy of ovarian masses (OMs) from transvaginal ultrasonography (TUS) andserum CA-125. | | |  |
| Methods | **Country** | Italy |  |
|  | **Funding** | No |  |
|  | **Design** | Retrospective, and prospective case-control |  |
| Participants | **Population description** | See inclusion and exclusion criteria |  |
|  | **Inclusion criteria** | Cohort (1) (1) Age (years) ≥ 18 years underwent TUS and were diagnosed with OMs Cohort (2) (1) Age (years) ≥ 18 years underwent TUS and were diagnosed with OMs CA125 (U/ML) test |  |
|  | **Exclusion criteria** | N/A |  |
|  | **Number** | 274 Retrospective cohort 239 ; Prospective cohort 35 |  |
| Model | **Imaging technique** | 2D ultrasound |  |
|  | **CAD model** | VGGNet ((d)CNN = (deep) Convolutional Neural Network);  ResNet ((d)CNN = (deep) Convolutional Neural Network);  MobileNet ((d)CNN = (deep) Convolutional Neural Network) DSS |  |
|  | **Features** | 269 solid ; 278 liquid ; 306 mixed solid  CA125 (U/ML) ; pre/postmenopausal status  Acoustic shadow  Age (years) |  |
|  | **Test(s) used to select significant features** | N/A |  |
|  | **Evaluation setup** | only mentioned in the abstract, most likely 10-fold cross validation as  External validation in prospective cohort (n=35) |  |
|  | **ROI (region of interest) annotated** | Manually; automatically |  |
| Results | **Samples (n)** | 274  Retrospective 239 123 benign; 116 malignant  Prospective 35 15 benign; 20 malignant |  |
|  | **Age (years)** | Retrospective cohort 18 - 84, mean 55  Prospective cohort 18 -73, mean 50 |  |
|  | **CA125 (U/ML)** | Premenopausal 200 U/ml  Postmenopausal > 71 U/ml  Normal 30U/ml |  |
|  | **Menopausal status** | Retrospective cohort Premenopausal 95 Postmenopausal 144  Prospective cohort Premenopausal 15 Postmenopausal 20 |  |
|  | **Timeframe scanning for surgery** | 2 weeks |  |
|  | **Timeframe data collection** | Retrospective cohort: Jan 2017 - Dec 2019 Prospective cohort: Jan 2021 - April 2021 |  |
|  | **Outcome** | Accuracy  AUC  Sensitivity  Specificity  PPV NPV |  |
|  | **Intervention compared to** | Histology and 2 gynecologists (2 and 10 years’ experience) |  |

| **Christiansen *et al.* 2021 (#9)^36^** | | | |
| --- | --- | --- | --- |
| **Title** Ultrasound image analysis using deep neural networks for discriminating between benign and malignant ovarian tumors: comparison with expert subjective assessment | | |  |
| **Objective** (1) to develop and test a DNN-based computerized ultrasound image analysis model for discriminating between benign and malignant ovarian tumors (2) to compare its diagnostic accuracy with that of expert subjective assessment (SA) and IOTA (International Ovarian Tumor Analysis) simple rules and simple-rules risk. | | |  |
| Methods | **Country** | Sweden |  |
|  | **Funding** | N/A |  |
|  | **Design** | Retrospective Case-control |  |
| Participants | **Population description** | See inclusion and exclusion criteria |  |
|  | **Inclusion criteria** | (1) surgery within 120 days after US examination  (2) follow-up for a minimum of 3 years or until resolution the ultrasound examination. |  |
|  | **Exclusion criteria** | N/A |  |
|  | **Number** | 758 (1) n=634 (surgery) (2) n=124 (follow-up) |  |
| Model | **Imaging technique** | Grayscale and power doppler |  |
|  | **CAD model** | VGGNet ((d)CNN = (deep) Convolutional Neural Network); ResNet ((d)CNN = (deep) Convolutional Neural Network); MobileNet ((d)CNN = (deep) Convolutional Neural Network) together Overy-Dx1 and Ovry-Dx2 |  |
|  | **Features** | Ovry-Dx1  The probability threshold for Ovry-Dx1 was intended to be set so as to give an optimal balance between sensitivity and specificity, with a sensitivity close to that of SA. Since this value was near 0.5 and its uncertainty large, the threshold was simply set to 0.5, with cases above this threshold classified as malignant  Ovry-Dx2  For Ovry-Dx2, the probability thresholds were set to 0.4 and 0.6 (i.e. cases with a predicted probability of malignancy between 0.4 and 0.6 were classified as inconclusive), which was shown to result in a reasonable balance between performance and fraction of excluded cases in the validation set  1024 layers for ResNet  512 layers MobileNet |  |
|  | **Test(s) used to select significant features** | N/A |  |
|  | **Evaluation setup** | a training set (n=508) 67%; a validation set (n=100) 13%; a test set (n=150) 20% |  |
|  | **ROI (region of interest) annotated** | Manually, sonographist |  |
| Results | **Samples (n)** | 3077 1927 grayscale + 1150 power doppler  449 benign; 309 malignant |  |
|  | **Age (years)** | N/A |  |
|  | **CA125 (U/ML)** | N/A |  |
|  | **Menopausal status** | N/A |  |
|  | **Timeframe scanning for surgery** | 3 months |  |
|  | **Timeframe data collection** | 2010 - 2019 |  |
|  | **Outcome** | Sensitivity  Specificity  AUC  Accuracy |  |
|  | **Intervention compared to** | Histology and subjective assessment and other models |  |

| **Qi *et al.* 2021 (#21)^37^** | | | |
| --- | --- | --- | --- |
| **Title** Diagnosis of ovarian neoplasms using nomogram in combination with ultrasound Image-Base Radiomics signature and clinical factors | | |  |
| **Objective** This study utilizes a two-step radiomics classification of serous ovarian tumors based on the imaging and builds a nomogram combining the clinical factors to distinguish benign, borderline, and malignant ovarian tumors**.** | | |  |
| Methods | **Country** | China |  |
|  | **Funding** | National Natural Science Foundation of China science and Technology Development Fund of Tianjin Education Commission for Higher Education |  |
|  | **Design** | Retrospective case control |  |
| Participants | **Population description** | Women with serous ovarian tumors |  |
|  | **Inclusion criteria** | 1) histological diagnosis of benign, borderline, and malignant ovarian serous tumors; 2) availability of preoperative US images suitable for diagnostic analysis; 3) US scanning performed before neoadjuvant therapy or surgical resection. |  |
|  | **Exclusion criteria** | 1) no US results or the ovarian mass was not completely visible in the image;  2) mucinous, clear cell, endometrioid, metastatic cancer |  |
|  | **Number** | 265 |  |
| Model | **Imaging technique** | N/A |  |
|  | **CAD model** | Nomogram using LASSO and RADscore |  |
|  | **Features** | 855 features MATLAB  Age (years) CA125 (U/ML) level lesion location Family history of cancer Ascites Radscore  2 models were build  1) clinical feature model 2) combined clinical feature model (CCR)  to distinguish two tasks 1) benign vs borderline and malignant 2) borderline vs malignant  Task1 17 features  Task2 22 features Sonographists 2x (junior / senior) on validation set |  |
|  | **Test(s) used to select significant features** | Wilson test Pearson test Chi-square test Fischer's exact test Wilcoxon sum-rank Univariate analysis Binary univariate analysis (multivariate analysis) |  |
|  | **Evaluation setup** | 196 training 70% 83 validation 30% 10-fold cross-validation |  |
|  | **ROI (region of interest) annotated** | Manually, sonographist |  |
| Results | **Samples (n)** | 279  106 benign tumors 65 borderline tumors 108 malignant tumors  28 from 14 patients were bilateral (7 borderline / 7 serous OC) |  |
|  | **Age (years)** | Task 1 Training 51.2 ± 13.4 - benign 48.0 ± 13.5 - borderline / malignant  Test 49.1 ± 16.1- benign 49.7 ± 11.2 - borderline / malignant  Task 2 Training 43.8 ± 14.0 - borderline 52.3 ± 9.06 - malignant  Test 36.7 ± 13.0 - borderline 53.7 ± 12.2 - malignant |  |
|  | **CA125 (U/ML)** | computed as 0 and 1 0 ≤500 IU/L 1 >500 IU/L  Task 1 Training 0 and 1 75 (98.7) and 1 (1.3) - benign 73 (0.6) and 47 (0.4) - borderline / malignant Test 0 and 1 0 (0.0) and 30 (100.0) - benign 26 (49.1) and 27 (50.9) - borderline / malignant  Task 2 Training 0 and 1 35 (77.8) and 10 (22.2) - borderline 35 (45.4) and 42 (54.5) - malignant Test 0 and 1 6 (30.0) and 15 (48.4) - borderline 15 (48.4) and 16 (51.6) - malignant |  |
|  | **Menopausal status** | N/A |  |
|  | **Timeframe scanning for surgery** | N/A |  |
|  | **Timeframe data collection** | Mar 2013 - Dec 2016 |  |
|  | **Outcome** | AUC Sensitivity Specificity  Accuracy |  |
|  | **Intervention compared to** | Histology and sonographist (junior (2yrs) / senior 8 yrs) validation set |  |

| **Stefan *et al.* 2021 (#26)^63^** | | | |
| --- | --- | --- | --- |
| **Title** Ultrasonography in the Diagnosis of Adnexal Lesions: The Role of Texture Analysis | | |  |
| **Objective** Ultrasonography in the Diagnosis of Adnexal Lesions: The Role of Texture Analysis | | |  |
| Methods | **Country** | Romania |  |
|  | **Funding** | No |  |
|  | **Design** | Retrospective case-control |  |
| Participants | **Population description** |  |  |
|  | **Inclusion criteria** | (1) a lesion with a minimum diameter of at least 20 mm, (2) the availability of conventional B-mode images, (3) lack of imaging artifacts, (4) the existence of a patient’s serial number (PSN) |  |
|  | **Exclusion criteria** | (1) no medical data corresponding to the PSN (2) the absence of a final pathological diagnosis to indicate the benign or malignant nature of the lesions (3) the pathological analysis performed at more than 30 days after the image acquisition (4) no gynecological follow-up |  |
|  | **Number** | 120 |  |
| Model | **Imaging technique** | 2D ultrasound |  |
|  | **CAD model** | k-nearest neighbor KNNs |  |
|  | **Features** | 26 features to 23 to 3 features CNN used for denoising image (1) histogram analysis (2) gradient (3) run-length matrix (RLM) (4) gray-level co-occurrence matrix (GLCM) (5) autoregressive model (6) wavelet transformation the MaZda software with B11 program |  |
|  | **Test(s) used to select significant features** | (1) The Mann– Whitney U test (Univariate) (2) Multiple regression analysis ROC-curves (3) Intraclass coefficient (IC) the Bonferroni correction |  |
|  | **Evaluation setup** |  |  |
|  | **ROI (region of interest) annotated** | Semi-automatic with manual correction, researcher |  |
| Results | **Samples (n)** | 123 images 85 benign 35 malignant |  |
|  | **Age (years)** | 22–76 years (averAge (years) Age (years) ± standard deviation: 38.15 ± 14.68 years) |  |
|  | **CA125 (U/ML)** | N/A |  |
|  | **Menopausal status** | N/A |  |
|  | **Timeframe scanning for surgery** | N/A |  |
|  | **Timeframe data collection** | Oct 2017- Feb 2019 |  |
|  | **Outcome** | Sensitivity Specificity  Accuracy  PPV  NPV |  |
|  | **Intervention compared to** | histology |  |

| **Wang *et al.* 2021 (#10)^38^** | | | |
| --- | --- | --- | --- |
| **Title** Application of Deep Convolutional Neural Networks for Discriminating Benign, Borderline, and Malignant Serous Ovarian Tumors From Ultrasound Images | | |  |
| **Objective** To evaluate the performance of the deep convolutional neural network (DCNN) to discriminate between benign, borderline, and malignant serous ovarian tumors (SOTs) on ultrasound(US) images**.** | | |  |
| Methods | **Country** | China |  |
|  | **Funding** | National Natural Science Foundation of China |  |
|  | **Design** | Retrospective Case-control |  |
| Participants | **Population description** | Patients with a serous ovarian tumor |  |
|  | **Inclusion criteria** | (1) a histologic diagnosis of benign, borderline, or malignant SOTs  (2) availability of diagnostic-quality preoperative US images  (3) US scanning before neoadjuvant therapy or surgical resection. |  |
|  | **Exclusion criteria** | (1) no ultrasound results or the ovarian mass was not completely in the images (2) no mucinous, clear cell, endometrioid tumors (3) metastatic cancer. |  |
|  | **Number** | 265 |  |
| Model | **Imaging technique** | N/A |  |
|  | **CAD model** | VGGNet ((d)CNN = (deep) Convolutional Neural Network);  GoogLeNet ((d)CNN = (deep) Convolutional Neural Network);  ResNet ((d)CNN = (deep) Convolutional Neural Network);  MobileNet ((d)CNN = (deep) |  |
|  | **Features** | Number of features unclear  2-step and 3 step classification  2 step is benign vs borderline/malignant and 3 step is benign vs borderline vs malignant  Preprocessing Python Randomly assigned to training 70% and validation 30% - 500 epochs learning rate decayed every 20 epochs - We then fine-tuned the parameters of the fully connected layer of the network on our dataset via back propagation - Class Activation Mapping (CAM) is a fully trained network to make localization map is completely generated by the fully trained network without additional manual annotation  Task A = benign vs malignant Task B = borderline vs malignant Task C = benign vs borderline vs malignant - |  |
|  | **Test(s) used to select significant features** | N/A |  |
|  | **Evaluation setup** | Transfer learning used - 3-fold-crossvalidation |  |
|  | **ROI (region of interest) annotated** | Manually and automatically |  |
| Results | **Samples (n)** | 279 images 108 benign; 65 borderline; 106 malignant |  |
|  | **Age (years)** | 15-79 |  |
|  | **CA125 (U/ML)** | N/A |  |
|  | **Menopausal status** | N/A |  |
|  | **Timeframe scanning for surgery** | N/A |  |
|  | **Timeframe data collection** | Mar 2013 – Dec 2016 |  |
|  | **Outcome** | Sensitivity Specificity  Accuracy  F1-score AUC |  |
|  | **Intervention compared to** | Histology and sonographist (12 years experience) |  |

| **Martinez-Mas *et al.* 2019 (#50)**^39^ | | | |
| --- | --- | --- | --- |
| **Title** Evaluation of machine learning methods with Fourier Transform features for classifying ovarian tumors based on ultrasound images | | |  |
| **Objective** To find the best classifier using the FT, and as a novelty, the ELM algorithm has been used and compared with classical classifiers in this type of problem | | |  |
| Methods | **Country** | Spain |  |
|  | **Funding** | Spanish MINECO under grant National Institute for Health Research Biomedical Research Centre |  |
|  | **Design** | Retrospective case control |  |
| Participants | **Population description** |  |  |
|  | **Inclusion criteria** |  |  |
|  | **Exclusion criteria** |  |  |
|  | **Number** | 187 |  |
| Model | **Imaging technique** | B-mode without Doppler |  |
|  | **CAD model** | SVM; KNN; LD;ELM |  |
|  | **Features** | Two types of feature vectors were originally extracted  (1) Histograms of Intensity Features  (2) Local Binary Pattern Features within 4 settings:  (1) Original image (2) Enhanced image (3) Segmented Region of Interest  (4) Segmented Region of Interest Enhanced Best classification performance could be reached by means of features computed on the Segmented ROI Enhanced.  Next Fourier Transform features are computed for evaluating ML models by making use of FFT (Fast Fourier Transform) and DFT (Discrete Fourier Transform).  Number of features unclear |  |
|  | **Test(s) used to select significant features** | N/A |  |
|  | **Evaluation setup** | Leave-One-Out Cross Validation (LOO-CV) procedure N=30 |  |
|  | **ROI (region of interest) annotated** | Manually, unclear by whom |  |
| Results | **Samples (n)** | 384 ultrasound images 112 benign 75 malignant |  |
|  | **Age (years)** | N/A |  |
|  | **CA125 (U/ML)** | N/A |  |
|  | **Menopausal status** | N/A |  |
|  | **Timeframe scanning for surgery** | 4 months |  |
|  | **Timeframe data collection** | N/A |  |
|  | **Outcome** | Sensitivity Specificity  Accuracy AUC |  |
|  | **Intervention compared to** | Histology |  |

| **Zhang *et al.* 2019 (#54)^40^** | | |
| --- | --- | --- |
| **Title** Improved Deep Learning Network Based in combination with Cost-sensitive Learning for Early Detection of Ovarian Cancer in Color Ultrasound Detecting System | | |
| **Objective** (1) Presenting an image diagnosis system for classifying the ovarian cysts in color ultrasound images, which novelly applies the image features fused by both high-level features from deep learning network and low-level features from texture descriptor. (2) To improve the accuracy of early diagnosis of ovarian cancer with improved convolutional neural network | | |
| Methods | **Country** | China |
|  | **Funding** | N/A |
|  | **Design** | Retrospective case control |
| Participants | **Population description** | Ultrasound and histology samples of ovarian tumors (open source database) |
|  | **Inclusion criteria** | N/A |
|  | **Exclusion criteria** | N/A |
|  | **Number** | N/A |
| Model | **Imaging technique** | N/A (open source database) |
|  | **CAD model** | RF, VGGNet, GoogleNet, FCNN = Fully Connected Convolutional Neural Network  CNN = Five-Layer Connected Convolutional Neural Network  AlexNet, RF = cost-sensitive RF |
|  | **Features** | Fusion of deep feature and texture for ovarian cysts Data-augmentation was performed: 1628 ovarian ultrasound images from different case are randomly grouped and expanded seven times Each grouping ensured that 806 of them are used to train GoogLeNet 420 images are used to validate GoogLeNet model Softmax Loss function Weights given to features ULBP All introduced into cost-sensitive RF (1) Bootstrap data set is acquired through Bagging method (2) Establishing non-pruning classification regression (CART) decision tree for each Bootstrap data set, and introduce cost factor into Gini index calculation of CART decision tree |
|  | **Test(s) used to select significant features** | N/A |
|  | **Evaluation setup** | Transfer Learning number of decision tree 10  10-fold cross validation  Training 306 71.5% Validation 61 14.3%  Test set 61 (external validation) 14.3% |
|  | **ROI (region of interest) annotated** | N/A |
| Results | **Samples (n)** | Pedraza *et al.*/ 428 US images 357 malignant 71 benign Peking Union hospital 1400 US images 277 malignant 299 benign |
|  | **Age (years)** | N/A |
|  | **CA125 (U/ML)** | N/A |
|  | **Menopausal status** | N/A |
|  | **Timeframe scanning for surgery** | N/A |
|  | **Timeframe data collection** | N/A |
|  | **Outcome** | Sensitivity Specificity  Accuracy AUC |
|  | **Intervention compared to** | Histology |

| **Acharya *et al.* 2018 (#183)^41^** | | | |
| --- | --- | --- | --- |
| **Title** Use of Nonlinear Features for Automated Characterization of Suspicious Ovarian Tumors Using Ultrasound Images in FuzzyForest Framework | | |  |
| **Objective** (1) We investigate the use of the popular random forest classifier, to boost measures in of sensitivity, specificity and accuracy. (2) We investigated the role of fuzzy forests ensemble classifier | | |  |
| Methods | **Country** | China |  |
|  | **Funding** | N/A |  |
|  | **Design** | Cohort study |  |
| Participants | **Population description** | Women with ovarian mass before surgery |  |
|  | **Inclusion criteria** | N/A |  |
|  | **Exclusion criteria** | N/A |  |
|  | **Number** |  |  |
| Model | **Imaging technique** | B-mode ultrasonography, 2D color doppler, 3D transvaginal ultrasonography |  |
|  | **CAD model** | KNN, RF, Fuzzy Forest, FRNN |  |
|  | **Features** | 810 features (non-linear feature extraction)  796 features (non-significant) using  WGCNA 39 features after using Relief-F algorithm was tested  469 |  |
|  | **Test(s) used to select significant features** | WGCNA = weighted correlation network analysis Relief-F = recursive feature elimination random forest PCA = principle component analysis |  |
|  | **Evaluation setup** | 10-fold cross validation |  |
|  | **ROI (region of interest) annotated** | Manually, radiologist and gynecologist |  |
| Results | **Samples (n)** | 469 238 suspicious 281 non-suspicious |  |
|  | **Age (years)** | 23-90 |  |
|  | **CA125 (U/ML)** | N/A |  |
|  | **Menopausal status** | N/A |  |
|  | **Timeframe scanning for surgery** | N/A |  |
|  | **Timeframe data collection** | N/A |  |
|  | **Outcome** | Sensitivity Specificity  Accuracy AUC |  |
|  | **Intervention compared to** | Non-fuzzy versus fuzzy classifier |  |

| **Aramendia-Vidaurreta *et al.* 2016 (#183)^46^** | | | |
| --- | --- | --- | --- |
| **Title** Ultrasound Image Discrimination between Benign and Malignant Adnexal Masses Based on a Neural Network Approach | | |  |
| **Objective** To develop a CAD technique for ultrasound images to be able to discriminate between benign and malignant adnexal masses and based on neural networks capable of over-coming the limitations of the available approaches**.** | | |  |
| Methods | **Country** | Spain |  |
|  | **Funding** | N/A |  |
|  | **Design** | Case-control |  |
| Participants | **Population description** | Women with adnexal mass |  |
|  | **Inclusion criteria** | N/A |  |
|  | **Exclusion criteria** | N/A |  |
|  | **Number** | 145 |  |
| Model | **Imaging technique** | 2D transvaginal ultrasound |  |
|  | **CAD model** | MLP Multilayer Perceptron Networks |  |
|  | **Features** | Started with 80 features 40 significant features  Age (years); 4 LBP hist variance; 2 LBP entropy; 3 LBP avgPower; 2 entropy; 2 invariant moments; 3 GLCM; 23 GW  To select features were used  (1) LBP = local binary pattern (2) Entropy - Kapur  (3) Hu's invariant moments (4) GLCM (5) Laws texture image  (6) GWT (7) CGP |  |
|  | **Test(s) used to select significant features** | Student's t-test |  |
|  | **Evaluation setup** | 80% testing 10% validation 10% testing  10x cross validation |  |
|  | **ROI (region of interest) annotated** | Manually, unclear by whom |  |
| Results | **Samples (n)** | 145 images 106 benign 39 malignant |  |
|  | **Age (years)** | 35–65 years, mean 43 |  |
|  | **CA125 (U/ML)** | N/A |  |
|  | **Menopausal status** | N/A |  |
|  | **Timeframe scanning for surgery** | N/A |  |
|  | **Timeframe data collection** | N/A |  |
|  | **Outcome** | Sensitivity Specificity  Accuracy AUC  PPV |  |
|  | **Intervention compared to** | Histology |  |

| **Khazendar *et al.* 2015 (#472)^47^** | | | |
| --- | --- | --- | --- |
| **Title** Automated characterization of ultrasound images of ovarian tumors: the diagnostic accuracy of a support vector machine and image processing with a local binary pattern operator | | |  |
| **Objective** To develop a computerized system, capable of characterizing images of ovarian masses as benign or malignant independent of an examiner being competent to identify the features required to make a diagnosis. | | |  |
| Methods | **Country** | United Kingdom, Belgium |  |
|  | **Funding** | Ministry of the Higher Education in Kurdistan |  |
|  | **Design** | Retrospective cohort study |  |
| Participants | **Population description** | Women recruited in the IOTA study (2010) whom underwent surgical removal of the mass |  |
|  | **Inclusion criteria** | N/A |  |
|  | **Exclusion criteria** | N/A |  |
|  | **Number** | 177 |  |
| Model | **Imaging technique** | 2D B-mode |  |
|  | **CAD model** | SVM and LBP |  |
|  | **Features** | LBP with 8 bit binary code with decimal value 0 – 255 1024 feature components in one feature vector image, with R = 2 Imbalance problem benign versus malignant randomly sampled 50 benign and 50 malignant totaling  100 images for training and testing |  |
|  | **Test(s) used to select significant features** | N/A |  |
|  | **Evaluation setup** | 50-fold cross validation performance of the SVM per 15 cycles |  |
|  | **ROI (region of interest) annotated** | Manually, sonographist and student |  |
| Results | **Samples (n)** | 187  112 benign 75 malignant |  |
|  | **Age (years)** | N/A |  |
|  | **CA125 (U/ML)** | N/A |  |
|  | **Menopausal status** | N/A |  |
|  | **Timeframe scanning for surgery** | N/A |  |
|  | **Timeframe data collection** | Nov 2005 - Nov 2013 |  |
|  | **Outcome** | Sensitivity Specificity  Accuracy |  |
|  | **Intervention compared to** | Histology |  |

| **Acharya *et al.* 2014 (#66)^42^** | | | |
| --- | --- | --- | --- |
| **Title** GyneScan: An Improved Online Paradigm for Screening of Ovarian Cancer via Tissue Characterization | | |  |
| **Objective** To propose an effective adjunct CAD technique called GyneScan for ovarian tumor detection in ultrasound images | | |  |
| Methods | **Country** | China, United States of America, United Kingdom, Greece |  |
|  | **Funding** | N/A |  |
|  | **Design** | Retrospective Case-control |  |
| Participants | **Population description** | N/A |  |
|  | **Inclusion criteria** | Previous diagnosis of ovarian mass and presurgical |  |
|  | **Exclusion criteria** | Women with no anatomopathological evaluation |  |
|  | **Number** | 20 |  |
| Model | **Imaging technique** | B-mode 2D power doppler 3D transvaginal |  |
|  | **CAD model** |  |  |
|  | **Features** | The middle from each of the 10 benign and 10 malignant subjects, thus making our database to have 1300 malignant and 1300 benign images was selected. Feature extraction with (1) Gray Level Co-occurrence Matrix (GLCM) (2) Ru length matrix  Started with 42 features ended with 40 features, per classifier was a number of features selected.  SVM types used (1) linear kernel (2) quadratic kernel (3) polynomial kernel of order 1, 2, and 3 (4) Radial Basis Function (RBF)  (1) Maximum Relevance Minimum Redundancy (mRMR) (2) Mutual Information Quotient (MIQ) method as the feature selection method Depending on the classifier a number of features was selected 11:  (1) Short Run Emphasis (SRE); (2) Long Run Emphasis (LRE); (3) Gray-level Non-uniformity (GLNU); (4) Run length Non-uniformity (RLNU); (5) Run percentage) (RP); (6) Low Gray-level Run Emphasis (LGRE); (7) High Gray-level Run Emphasis (HGRE); (8) Short Run Low Gray-level Run Emphasis (SRLGRE); (9) Short Run High Gray-level Run Emphasis (SRHGRE); (10) Long Run Low Gray-level Run Emphasis (LRLGE); (11) Long Run High Gray-level Run Emphasis (LRHGE) |  |
|  | **Test(s) used to select significant features** | Students t-test |  |
|  | **Evaluation setup** | Training and testing set  10 fold cross validation |  |
|  | **ROI (region of interest) annotated** | Manually, radiologist and gynecologist |  |
| Results | **Samples (n)** | 10 benign; 10 malignant 2600 images 1300 benign 1300 malignant |  |
|  | **Age (years)** | 29 - 74 |  |
|  | **CA125 (U/ML)** | N/A |  |
|  | **Menopausal status** | 11 pre-menopausal 9 post-menopausal |  |
|  | **Timeframe scanning for surgery** | N/A |  |
|  | **Timeframe data collection** | N/A |  |
|  | **Outcome** | Sensitivity  Specificity  Accuracy |  |
|  | **Intervention compared to** | Histology |  |

| **Acharya *et al.* 2014 (#216)^43^** | | | |
| --- | --- | --- | --- |
| **Title** Evolutionary algorithm-based classifier parameter tuning for automatic ovarian cancer tissue characterization and classification. | | |  |
| **Objective** To present one such CAD technique for ovarian tumor classification and to evaluate the performance of the technique using training and test ovarian tumor image databases. | | |  |
| Methods | **Country** | China |  |
|  | **Funding** | N/A |  |
|  | **Design** | Prospective cohort study |  |
| Participants | **Population description** | Women with adnexal masses |  |
|  | **Inclusion criteria** | See population description |  |
|  | **Exclusion criteria** | See population description |  |
|  | **Number** | 20 |  |
| Model | **Imaging technique** | B-mode ultrasound 2D doppler 3D transvaginal ultrasound |  |
|  | **CAD model** | Probabilistic Neural Network (PNN) |  |
|  | **Features** | - 23 features 1. Hu's invariant moments (4) 2. Gabor wavelet transform (17) 3. Yager and Kapur entropies (2)  - 10 sets with each 260 images. 9 sets (N=2340) 90% are used for training and 1 set (N=260) 10 %is used for testing  - 10-fold cross validation |  |
|  | **Test(s) used to select significant features** | Student’s t-test |  |
|  | **Evaluation setup** | 9 sets (2340 images) are used for training and 1 set (260 images) is used for testing  10 fold cross validation |  |
|  | **ROI (region of interest) annotated** | Manually by radiologist and gynecologist |  |
| Results | **Samples (n)** | 20  10 benign  10 malignant  2600 images; 1300 benign; 1300 malignant |  |
|  | **Age (years)** | Mean 49.5 (29-74) |  |
|  | **CA125 (U/ML)** | N/A |  |
|  | **Menopausal status** | 11 premenopausal 9 postmenopausal |  |
|  | **Timeframe scanning for surgery** | N/A |  |
|  | **Timeframe data collection** | N/A |  |
|  | **Outcome** | Accuracy  Sensitivity  Specificity  PPV |  |
|  | **Intervention compared to** | N/A |  |
| **Acharya *et al.* 2013 (#70)^45^** | | | |
| **Title** Ovarian tumor characterization and classification using ultrasound-a new online paradigm | | |  |
| **Objective** To develop an adjunct computer-aided diagnostic technique that uses 3D ultrasound images of the ovary to accurately characterize and classify benign and malignant ovarian tumors**.** | | |  |
| Methods | **Country** | China, United States of America, Malaysia |  |
|  | **Funding** | N/A |  |
|  | **Design** | Prospective case-control study |  |
| Participants | **Population description** | Pre/postmenopausal women consecutively selected for surgery with an adnexal mass |  |
|  | **Inclusion criteria** | See population description |  |
|  | **Exclusion criteria** | Patients with no anatomopathological evaluation |  |
|  | **Number** | 20 |  |
| Model | **Imaging technique** | 3D-transvaginal ultrasonography |  |
|  | **CAD model** | Decision Tree (DT) |  |
|  | **Features** | - 729 features (5 texture-based and 724 HOS-based) HOS = high order spectra GLCM = Gray-level Co-occurence Matrix FD = fraction Dimension - 4 features significant |  |
|  | **Test(s) used to select significant features** | Student's t-test |  |
|  | **Evaluation setup** | Training and testing set  10-fold cross validation |  |
|  | **ROI (region of interest) annotated** | Whole ultrasound image is used 🡪 so no use of ROI |  |
| Results | **Samples (n)** | 20  10 benign; 10 malignant  2000 images ; 1000 benign ; 1000 malignant |  |
|  | **Age (years)** | Mean 49.5 (29-74) |  |
|  | **CA125 (U/ML)** | N/A |  |
|  | **Menopausal status** | 11 premenopausal 9 postmenopausal |  |
|  | **Timeframe scanning for surgery** | N/A |  |
|  | **Timeframe data collection** | N/A |  |
|  | **Outcome** | Sensitivity  Specificity  PPV  NPV  TP rate  FP rate  TN rate  FN rate |  |
|  | **Intervention compared to** | Histology |  |
| **Faschingbauer *et al.* 2013 (#72)^48^** | | | |
| **Title** Automatic texture-based analysis in ultrasound imaging of ovarian masses | | |  |
| **Objective** (1) To assess the diagnostic accuracy of a newly developed automatic texture-based algorithm (ATBA) in ultrasound imaging of ovarian masses  (2) To compare its performance to subjective assessment by examiners with different levels of ultrasound experience. | | |  |
| Methods | **Country** | Germany |  |
|  | **Funding** | N/A |  |
|  | **Design** | Retrospective cross sectional |  |
| Participants | **Population description** | See inclusion criteria |  |
|  | **Inclusion criteria** | Preoperative transvaginal ultrasound |  |
|  | **Exclusion criteria** | N/A |  |
|  | **Number** | 105 |  |
| Model | **Imaging technique** | Ultrasound |  |
|  | **CAD model** | SVM |  |
|  | **Features** | - Feature extraction by ATBA (automatic, texture-based analysis), also with SFG (statistical geometric features)  - 16 numerical features build with SFG and stored in a automatic SVM, software package rapid miner. |  |
|  | **Test(s) used to select significant features** | Cohen's Kappa diagnostic performance among group of examiners  2-sided one-sample t-test  Youden's index |  |
|  | **Evaluation setup** | 1-fold cross-validation approach was used to separate training from testing data. Training and testing set |  |
|  | **ROI (region of interest) annotated** | Manually |  |
| Results | **Samples (n)** | 105  70 benign  35 malignant |  |
|  | **Age (years)** | 15–84 years  Median 42 |  |
|  | **CA125 (U/ML)** | N/A |  |
|  | **Menopausal status** | 54 premenopausal  51 postmenopausal |  |
|  | **Timeframe scanning for surgery** | N/A |  |
|  | **Timeframe data collection** | Aug 2005 - Jun 2008 |  |
|  | **Outcome** | Accuracy  Sensitivity  Specificity  Youden-index |  |
|  | **Intervention compared to** | Histology and gynecologists |  |
| **Acharya *et al.* 2012 (#73)^44^** | | | |
| **Title** Ovarian Tumor Characterization using 3D Ultrasound | | |  |
| **Objective** (1) To propose a novel image mining CAD technique using texture features for the classification of benign and malignant ovarian tumors from ultrasound images. (2) To develop a novel integrated index formulated as a combination of the texture features. | | |  |
| Methods | **Country** | China, United States of America |  |
|  | **Funding** | Unclear |  |
|  | **Design** | Retrospective cohort study |  |
| Participants | **Population description** | Presurgical evaluation of women with adnexal mass |  |
|  | **Inclusion criteria** | See population description |  |
|  | **Exclusion criteria** | See population description |  |
|  | **Number** | 20 |  |
| Model | **Imaging technique** | B-mode 2D power doppler 3D transvaginal ultrasound |  |
|  | **CAD model** | SVM-RBF |  |
|  | **Features** | - Features were selected by using (1) LBP local binary patterns (2) LTE laws' texture Energy LBP energy 3 LTE energy 8 14 features SVM types used (1) linear kernel (2) quadratic kernel (3) polynomial kernel of order 1, 2, and 3 (4) Radial Basis Function (RBF) |  |
|  | **Test(s) used to select significant features** | Student’s t-test |  |
|  | **Evaluation setup** | 10 fold cross validation for 2000 images Training and test set |  |
|  | **ROI (region of interest) annotated** | Automatically |  |
| Results | **Samples (n)** | 20 patients  10 benign  10 malignant  2000 images  1000 benign  1000 malignant |  |
|  | **Age (years)** | Mean 49.5 (29 – 74) |  |
|  | **CA125 (U/ML)** | N/A |  |
|  | **Menopausal status** | 11 pre-menopausal  9 post-menopausal |  |
|  | **Timeframe scanning for surgery** | N/A |  |
|  | **Timeframe data collection** | N/A |  |
|  | **Outcome** | Accuracy  Sensitivity  Specificity  PPV  TP rate  FP rate  TN rate  FN rate |  |
|  | **Intervention compared to** | Unclear |  |
| **Vaes *et al.* 2012 (#75)^49^** | | | |
| **Title** Differential diagnosis of adnexal masses: sequential use of the risk of malignancy index and HistoScanning, a novel computer-aided diagnostic tool | | |  |
| **Objective** To assess the value of ovarian HistoScanning, a novel computerized technique for interpreting ultrasound data, in combination with the risk of malignancy index (RMI) in improving triage for women with adnexal masses. | | |  |
| Methods | **Country** | United Kingdom, Belgium |  |
|  | **Funding** | Unclear |  |
|  | **Design** | Prospective cross-sectional study |  |
| Participants | **Population description** | See inclusion and exclusion criteria |  |
|  | **Inclusion criteria** | (1) at least 18 years scheduled for surgical removal of one or two ovaries because of the presence of an adnexal mass or as part of a prophylactic oophorectomy or hysterectomy with oophorectomy for a uterine abnormality (fibroid tumor, endometrial cancer) (2) written informed consent |  |
|  | **Exclusion criteria** | (1) history of ovarian cancer, previous pelvic surgery, (2) chemotherapy for breast or ovarian cancer |  |
|  | **Number** | 197 |  |
| Model | **Imaging technique** | 2D transvaginal ultrasound  3D transvaginal ultrasound |  |
|  | **CAD model** | Ovarian Histoscanning (OVHS) combined with RMI3 |  |
|  | **Features** | - OVHS quantifies changes induced by malignant tissues in back- scattered ultrasound waves when applied to voxel data generated during TVS.  - 3 algorithms within OVHS applied  - The following scheme was adopted for this purpose: (1) patients with an RMI below a lower cut-off (LC) were considered to have a negative (non-malignant) test outcome (2) patients with an RMI ≥ an upper cut-off (UC) were considered to have a positive (malignant) test outcome (3) in between was HistoScanning was added RMI scores |  |
|  | **Test(s) used to select significant features** | ANOVA with F-test or the Kruskal–Wallis test  Chi-square |  |
|  | **Evaluation setup** | 70% training set 30% testing set  To test the variability in a subset, a random subsampling process was repeated 100 times so that 100 randomly selected training and testing sets were created |  |
|  | **ROI (region of interest) annotated** | Manually by sonographist |  |
| Results | **Samples (n)** | 291 adnexal masses  125 benign  166 malignant |  |
|  | **Age (years)** | Mean:  Normal 62  Benign 58  Malignant 57 |  |
|  | **CA125 (U/ML)** | Mean  17 (3-356) - normal  12 (4–487) - benign  2323 (7–32986) - malignant |  |
|  | **Menopausal status** | Postmenopausal  76% - benign  66% - malignant  89% - normal |  |
|  | **Timeframe scanning for surgery** | ‘shortly’ |  |
|  | **Timeframe data collection** | N/A |  |
|  | **Outcome** | Sensitivity  Specificity |  |
|  | **Intervention compared to** | Histology, RMI1 + OVHS, RMI2 + OVHS |  |
| **Vaes *et al.* 2011 (#81)^50^** | | | |
| **Title** Mathematical models to discriminate between benign and malignant adnexal masses: Potential diagnostic improvement using ovarian HistoScanning | | |  |
| **Objective** Whether a new technology, Ovarian HistoScanning, has an additional diagnostic value in mathematical models developed for the differential diagnosis of adnexal masses. | | |  |
| Methods | **Country** | United Kingdom, France |  |
|  | **Funding** | General Direction of Technologies, of Research, and of Energy of the Belgian Walloon Region |  |
|  | **Design** | Prospective case control study |  |
| Participants | **Population description** | See inclusion or exclusion criteria |  |
|  | **Inclusion criteria** | (1) women 18 years or older, scheduled for the surgical removal of 1 or 2 ovaries because of (a) a suspected adnexal mass or (b) prophylactic oophorectomy or as part of hysterectomy for a uterine pathology. (2) Only patients with CA-125 measurements between 199 of 383 |  |
|  | **Exclusion criteria** | (1) history of ovarian cancer, previous pelvic surgery, (2) chemotherapy for breast or ovarian cancer |  |
|  | **Number** | 197 |  |
| Model | **Imaging technique** | 3D transvaginal ultrasound |  |
|  | **CAD model** | Logistic regression, Neural network |  |
|  | **Features** | - In total 9 features: (1) CA125 (U/ML) (2) HSS = HistoScanning score (3) Sol (4) Flu (5) Pap (6) USS (7) Sept (8) Wall (9) Meno  - The model has 1 hidden layer containing 3 hidden units |  |
|  | **Test(s) used to select significant features** | Kruskal-Wallis test or Mann-Whitney U test  Spearman correlation coefficient  Akaike information corrected criterion (AICC) |  |
|  | **Evaluation setup** | - Training set 60% Test set 40%. The 60% training set split in 70% and 30% (randomly chosen)  - 100 bootstrap-resampled data sets were created from the LS with AICC selection 100 runs for the frequent final model and full model internal validation |  |
|  | **ROI (region of interest) annotated** | Manually by expert |  |
| Results | **Samples (n)** | 197 ultrasound images  365 ovarian tumors  77 - normal  125 - benign  166 - malignant |  |
|  | **Age (years)** | Mean  62 +/- 13 - normal  58 +/- 10 - benign  57 +/- 11 - malignant |  |
|  | **CA125 (U/ML)** | Median (range), U/mL  17 (3-356) - normal  12 (4–487) - benign  2323 (7–32986) - malignant |  |
|  | **Menopausal status** | Postmenopausal  89% - normal  76% - benign  66% - malignant |  |
|  | **Timeframe scanning for surgery** | N/A |  |
|  | **Timeframe data collection** | N/A |  |
|  | **Outcome** | AUC  Sensitivity  Specificity |  |
|  | **Intervention compared to** | Histology, RMI, LR1, LR2, NN1 and NN2 from Timmerman *et al.* |  |
| **Lucidarme *et al.* 2010 (#85)^52^** | | | |
| **Title** A new computer-aided diagnostic tool for non-invasive characterization of malignant ovarian masses: results of a multicentre validation study | | |  |
| **Objective** Prospectively assess a new way of increasing the ability of TVS to discriminate benign from malignant adnexal masses using a concept radically different from all tissue characterization technologies proposed so far: Ovarian HistoScanning. | | |  |
| Methods | **Country** | Belgium, Germany, Italy, Sweden, France and Israel |  |
|  | **Funding** | Unclear |  |
|  | **Design** | Prospective case control |  |
| Participants | **Population description** | See inclusion and exclusion criteria |  |
|  | **Inclusion criteria** | (1) least 18 years (2) scheduled for surgical removal of one or two ovaries because of the presence of an adnexal mass or as part of a prophylactic oophorectomy or hysterectomy with oophorectomy for a uterine abnormality (fibroid tumor, endometrial cancer) (3) written informed consent |  |
|  | **Exclusion criteria** | (1) history of ovarian cancer, previous pelvic surgery, (2) chemotherapy for breast or ovarian cancer |  |
|  | **Number** | 264 |  |
| Model | **Imaging technique** | 2D transvaginal ultrasound 3D transvaginal ultrasound |  |
|  | **CAD model** | Ovarian HistoScanning (OVHS) |  |
|  | **Features** | - OVHS which quantifies changes induced by malignant tissues in back- scattered ultrasound waves when applied to voxel data generated during TVS.  - 3 algorithms within OVHS applied |  |
|  | **Test(s) used to select significant features** | Unclear |  |
|  | **Evaluation setup** | One group |  |
|  | **ROI (region of interest) annotated** | Manually by gynecologists and sonographers |  |
| Results | **Samples (n)** | 375 ovaries  107 normal ovaries  127 benign  141 malignant  359 sonographers opinion  104 normal ovaries  119 benign  136 malignant |  |
|  | **Age (years)** | Mean  57 (26-86) |  |
|  | **CA125 (U/ML)** | ≥35 110 <35 94 |  |
|  | **Menopausal status** | 74 premenopausal  190 postmenopausal |  |
|  | **Timeframe scanning for surgery** | 1 day |  |
|  | **Timeframe data collection** | N/A |  |
|  | **Outcome** | PPV  NPV  TP rate  FP rate  TN rate  FN rate |  |
|  | **Intervention compared to** | Histology |  |
| **Lu *et al.* 2003 (#526)^51^** | | | |
| **Title** Preoperative prediction of malignancy of ovarian tumors using least squares support vector machines | | |  |
| **Objective** (1) To develop and (2) evaluate several least squares support vector machine (LS-SVM) classifiers within the Bayesian evidence framework, in order to preoperatively predict malignancy of ovarian tumors. | | |  |
| Methods | **Country** | Belgium |  |
|  | **Funding** | Unclear |  |
|  | **Design** | Case control study |  |
| Participants | **Population description** | See inclusion and exclusion criteria |  |
|  | **Inclusion criteria** | Patients with a persistent extra-uterine pelvic mass, which was subsequently surgically removed |  |
|  | **Exclusion criteria** | Missing CA125 (U/ML) value preoperatively |  |
|  | **Number** | 425 |  |
| Model | **Imaging technique** | Resistance Index Color score |  |
|  | **CAD model** | SVM |  |
|  | **Features** | - Bayesian evidence framework used to select hyperparameters for the LS-SVM  - 25 🡪 27 boxplot, rescaling (e.g. CA125 (U/ML) values)  - 10 variables / features with RBF-kernel (1) CA125 (U/ML) (log) (2) Papillations (3) Solid tumor (4) Normal blood flow (5) Bilateral mass (6) Post-menopausal (7) Abdominal fluid (8) Acoustic shadows (9) Strong blood flow (10) |  |
|  | **Test(s) used to select significant features** | Univariate analysis  Multivariate analysis  RBF kernel Linear kernels |  |
|  | **Evaluation setup** | - Irregular wall hold-out cross-validation 30 folds by splitting the dataset randomly into 2 sets  - Training set (N=265) 62% (1994 – 1997), test set (N=160) 38% (1997 - 1999). Malignant tumors both 1/3 in training and test set.  - External validation set 1 internal test set  1 external validation set |  |
|  | **ROI (region of interest) annotated** | N/A |  |
| Results | **Samples (n)** | 425  291 benign  134 malignant |  |
|  | **Age (years)** | Mean  52.4 (21–93) (1994 - 1997)  48.6 (16–78) (1997 - 1999) |  |
|  | **CA125 (U/ML)** | N/A |  |
|  | **Menopausal status** | Postmenopausal  48.3% (1994 - 1997)  41.9% (1997 - 1999) |  |
|  | **Timeframe scanning for surgery** | N/A |  |
|  | **Timeframe data collection** | 1994-1999 |  |
|  | **Outcome** | Accuracy  AUC  Sensitivity  Specificity  PPV NPV |  |
|  | **Intervention compared to** | Histology, RMI, LR1, LR2 |  |
| **Zimmer *et al.* 2003 (#312)^53^** | | | |
| **Title** An automatic approach for morphological analysis and malignancy evaluation of ovarian masses using B-scans | | |  |
| **Objective** An automatic technique for quantitative analysis and malignancy detection of ovarian masses using B-scan US images is presented. | | |  |
| Methods | **Country** | Israel |  |
|  | **Funding** | Israeli Ministry of Science and by the Abramson Center for Medical Physics. |  |
|  | **Design** | Retrospective case control |  |
| Participants | **Population description** | Women with adnexal mass |  |
|  | **Inclusion criteria** | N/A |  |
|  | **Exclusion criteria** | N/A |  |
|  | **Number** | 163 |  |
| Model | **Imaging technique** | 2D ultrasound |  |
|  | **CAD model** | Bayes method |  |
|  | **Features** | - Determination of ROI 4 boundary maps from original image (1) “morphologic” gradient (2) local coefficient of variation (3) local grey-level entropy (4) Hurst coefficient  - 256 x 4 = 1024 features  - Classification tumor type (1) SD of the grey level in the entire ROI (2) local CV (3) coefficient of variation (CV) in the entire ROI (4) the mean grey level in the entire  - ROI creating decision rules  - 4 features are final |  |
|  | **Test(s) used to select significant features** | Unclear |  |
|  | **Evaluation setup** | Training set (N=163) 85% External validation set (N = 28) 15% |  |
|  | **ROI (region of interest) annotated** | Manually by operator in 131/161 cases, in the others automatically |  |
| Results | **Samples (n)** | 163 images  25 transparent cyst  67 turbid cyst  50 significantly solid  21 solid |  |
|  | **Age (years)** | N/A |  |
|  | **CA125 (U/ML)** | N/A |  |
|  | **Menopausal status** | N/A |  |
|  | **Timeframe scanning for surgery** | N/A |  |
|  | **Timeframe data collection** | N/A |  |
|  | **Outcome** | Accuracy  Sensitivity  Specificity  PPV  NPV |  |
|  | **Intervention compared to** | Histology |  |

**Table 1b Characteristics of included studies per image modality CT (3)**

| **Li *et al.* 2022 (#1)^54^** | | | |
| --- | --- | --- | --- |
| **Title** Application Values of 2D and 3D Radiomics Models Based on CT Plain Scan in Differentiating Benign from Malignant Ovarian Tumors | | |  |
| **Objective** (1) To explore the performance of radiomic in differentiating benign from malignant ovarian incidental lesions (2) To compare the performances of 2D and 3D texture features | | |  |
| Methods | **Country** | China |  |
|  | **Funding** | Jianxi Province Department of Science and Technology |  |
|  | **Design** | Retrospective case control study |  |
| Participants | **Population description** | N/A |  |
|  | **Inclusion criteria** | 1) patients with ovarian tumor confirmed by histopathology 2) no history of malignant tumors other than ovarian tumor; 3) patients who were undergoing pelvic CT examination before surgery |  |
|  | **Exclusion criteria** | 1. patients whom received radiotherapy, chemotherapy, or radiotherapy- chemotherapy before CT examination 2) patients diagnosed with inflammatory diseases 3) patients with low image 2. quality |  |
|  | **Number** | 140 |  |
| Model | **Imaging technique** | Dual-source CT using automatically modulated scanning parameters: tube voltage 120 kV, tube current 150 mA, slice thickness 5 mm, reconstruction interval 1 mm, and slice internal 1 mm. |  |
|  | **CAD model** | 3D nomogram  2D nomogram |  |
|  | **Features** | 10 and 11 |  |
|  | **Test(s) used to select significant features** | Spearman rank correlation  Univariate logistic regression  Multivariate logistic regression  Mann-Whitney U test  Student's T-test |  |
|  | **Evaluation setup** 1 | internal validation set |  |
|  | **ROI (region of interest) annotated** | manually by imaging doctors |  |
| Results | **Samples (n)** | 140  62 benign  72 malignant |  |
|  | **Age (years)** | benign / malignant  <18 5 / 1  >18 ≤30 15 / 2  >30 ≤50 26 / 27  >50 18 / 46 |  |
|  | **CA125 (U/ml)** | benign / malignant  <35 31 / 8  >35 ≤200 30 / 21  >200, ≤500 2 / 15  >500 1 / 34 |  |
|  | **Menopausal status** | N/A |  |
|  | **Timeframe scanning for surgery** | 2 weeks |  |
|  | **Timeframe data collection** | Jul 2017 - Aug 2020 |  |
|  | **Outcome** | Accuracy  AUC  Sensitivity  Specificity |  |
|  | **Intervention compared to** | Histology |  |
| **Park *et al.* 2021 (#1)^55^** | | | |
| **Title** Decoding incidental ovarian lesions: use of texture analysis and machine learning for characterization and detection of malignancy | | |  |
| **Objective** (1) To compare CT texture features of benign and malignant ovarian lesions and (2) To build a machine learning model to detect malignancy in incidental ovarian lesions. | | |  |
| Methods | **Country** | United States of America |  |
|  | **Funding** | No |  |
|  | **Design** | Retrospective case control study |  |
| Participants | **Population description** | Women with an incidental ovarian lesion |  |
|  | **Inclusion criteria** | N/A |  |
|  | **Exclusion criteria** | 1) No CT-scan available 2) Preexisting malignant or benign lesion 3) Not incidental lesion (symptoms highly related to ovarian mass) 4) Inconclusive diagnosis of ovarian lesions (no pathology results) |  |
|  | **Number** | 427 |  |
| Model | **Imaging technique** | Contrast-enhanced CT of abdomen and pelvis following injection of 75â€“100 ml of an iodinated contrast agent (Omnipaque 350, Iohexol, GE Healthcare, Princeton, NJ, USA). Portal venous phase (70 s delay). Variation in the slice thickness (between 3 to 5 mm) |  |
|  | **CAD model** | Random forest (RF) |  |
|  | **Features** | 8 |  |
|  | **Test(s) used to select significant features** | Pearson correlation |  |
|  | **Evaluation setup** 1 | All samples |  |
|  | **ROI (region of interest) annotated** | manually by radiologist |  |
| Results | **Samples (n)** | 427  348 benign  79 malignant |  |
|  | **Age (years)** | 44 years (range, 19–87 yrs) - benign  61 years (range, 20–92) - malignant |  |
|  | **CA125 (U/ML)** | median, 15; range, 5–158 - benign (15/348)  median, 323; range, 7–4557 - malignant (34/79) |  |
|  | **Menopausal status** | N/A |  |
|  | **Timeframe scanning for surgery** | N/A |  |
|  | **Timeframe data collection** | Jan 2014 - Dec 2015 |  |
|  | **Outcome** | Accuracy  AUC  Sensitivity  Specificity |  |
|  | **Intervention compared to** | histology |  |
| **Li *et al.* 2021 (#1)^56^** | | | |
| **Title** A radiomics approach for automated diagnosis of ovarian neoplasm malignancy in computed tomography | | |  |
| **Objective** To develop a two-dimensional (2D) radiomics approach with computed tomography (CT) to differentiate between benign and malignant ovarian neoplasms. | | |  |
| Methods | **Country** | China |  |
|  | **Funding** | Yes, Funding of Guizhou Province Department of Science and Technology (No. Qiankehe Support and the Funding of Health Commission of Jiangxi Province. |  |
|  | **Design** | Retrospective case control study |  |
| Participants | **Population description** | N/A |  |
|  | **Inclusion criteria** | (1) female patients with histopathological verified ovarian tumors, (2) persons with no history of previous or cur- rent malignancy other than that of ovarian tumors, (3) patients who were subjected to preoperative high-resolution procedures for ovarian cancer staging, (4) patients who had preoperative CT for the pelvic area within the preceding half a month. |  |
|  | **Exclusion criteria** | (1) patients who were subjected before the CT examination to radiotherapy, chemotherapy or chemoradiotherapy (2) patients diagnosed to suffer from inflammatory disease conditions (3) patients with low-quality imaging records |  |
|  | **Number** | 160 |  |
| Model | **Imaging technique** | a tube voltage of 120 kVp, a tube current of 150 mAs, a section thickness of 5 mm, a reconstruction interval of 1 mm, and a slice gap of 1 mm. |  |
|  | **CAD model** | Nomogram |  |
|  | **Features** | 134 textural features for 134 ROIs 396 features for each ROI 3 steps performed for feature selection (1) replacing the outliers by the median of the same feature; (2) Z-score data normalization is applied.  Z-score normalization was done in the training dataset to eliminate the differences in the value scales of extraction features (1) Spearman (2) mRMR algorithm (3) LASSO method with 10-fold cross validation (4) RADscore  14 features |  |
|  | **Test(s) used to select significant features** | Spearman rank correlation test  Univariate logistic regression  Multivariate logistic regression  DCA decision analysis curve  Mann-Whitney U test  T-test  Chi-square |  |
|  | **Evaluation setup** | training n=95 71% test n=39 29% 134 training + validation  26 external validation  10-fold cross validation |  |
|  | **ROI (region of interest) annotated** | manually by radiologist |  |
| Results | **Samples (n)** | 160 images  134 training  62 benign  72 malignant  26 external validation set – details unclear |  |
|  | **Age (years)** | < 18 > 50 |  |
|  | **CA125 (U/ML)** | < 35 > 500 |  |
|  | **Menopausal status** | N/A |  |
|  | **Timeframe scanning for surgery** | N/A |  |
|  | **Timeframe data collection** | Jul 2017 - Jun 2019 |  |
|  | **Outcome** | Accuracy  AUC  Sensitivity  Specificity |  |
|  | **Intervention compared to** | Histology |  |

**Table 1c Characteristics of included studies per image modality MRI (6)**

| **Liu *et al* 2022 (#1)^57^** | | | |
| --- | --- | --- | --- |
| **Title** Two-dimensional and three-dimensional T2 weighted imaging-based radiomic signatures for the preoperative discrimination of ovarian borderline tumors and malignant tumors | | |  |
| **Objective** (1) To evaluate the diagnostic performance of the MRI radiomics model in discriminating ovarian BOTs from malignancies;  (2) To clarify whether three-dimensional MR-based radiomic signatures (of the whole lesion) could show better dis- criminative performance than two-dimensional radiomic signatures (of the maximum lesion) could in the same study sample. | | |  |
| Methods | **Country** | China |  |
|  | **Funding** | No |  |
|  | **Design** | Retrospective case control study |  |
| Participants | **Population description** | See inclusion and exclusion criteria |  |
|  | **Inclusion criteria** | (1) patients with no previous pelvic surgery; (2) patients with no previous gynaecological disease history; (3) patients who had MRI examinations performed at our institution before pelvic or laparoscopic surgery |  |
|  | **Exclusion criteria** | (1) patients with previous pelvic surgical history or radiation history; (2) patients whose MRI data were unavailable either due to the examination being performed at another institution or due to claustrophobia; (3) patients whose data lacked histological results |  |
|  | **Number** | 196 |  |
| Model | **Imaging technique** | Axial turbo spin-echo (TSE) T1-weighted imaging (T1WI), coronal TSE T2-weighted imaging (T2WI), and axial/sagittal TSE fat-suppressed T2WI (fs-T2WI). 2D MRI 3D MRI |  |
|  | **CAD model** | Radiomics segmentation model |  |
|  | **Features** | 396 radiomics features LASSO + Radscore Difference radiomics model versus 3D sagittal radiomics model In 2D segmentation, we chose one slice with the largest lesion diameter in two protocols as the premium picture for segmenting the whole lesion. In 3D segmentation, the entire lesion from both protocols was outlined and segmented slice by slice |  |
|  | **Test(s) used to select significant features** | Two sample t-test |  |
|  | **Evaluation setup** | Randomly divided into training cohort n=99 50% testing cohort n=97 50% 1 internal test set |  |
|  | **ROI (region of interest) annotated** | Manually by radiologist |  |
| Results | **Samples (n)** | 196  91 borderline  105 malignant |  |
|  | **Age (years)** | 39.8 ± 14.9 years borderline  51.9 ± 12.1 years malignant |  |
|  | **CA125 (U/ML)** | 553.32 ± 994.28 training set  300.30 ± 452.27 testing set |  |
|  | **Menopausal status** | N/A |  |
|  | **Timeframe scanning for surgery** | N/A |  |
|  | **Timeframe data collection** | Jan 2014 - Dec 2017 |  |
|  | **Outcome** | Accuracy  AUC  Sensitivity  Specificity  PPV NPV |  |
|  | **Intervention compared to** | Histology |  |
| **Song *et al.* 2021 (#1)^58^** | | | |
| **Title** Radiomics derived from dynamic contrast-enhanced MRI pharmacokinetic protocol features: the value of precision diagnosis ovarian neoplasms | | |  |
| **Objective** To evaluate the efficiency of 2- and 3-class classification predictive tasks constructed from radiomics features extracted from dynamic contrast-enhanced magnetic resonance imaging (DCE-MRI) pharmacokinetic (PK) protocol in discriminating among benign, borderline, and malignant ovarian tumors. | | |  |
| Methods | **Country** | China |  |
|  | **Funding** | Applied Basic Research Programs of Shanxi Province |  |
|  | **Design** | Prospective case control study |  |
| Participants | **Population description** | See inclusion and exclusion criteria |  |
|  | **Inclusion criteria** | Ultrasound findings suggestive of ovarian masses with solid components |  |
|  | **Exclusion criteria** | (1) contraindications to MRI scans or gadolinium contrast agents (2) insufficient tumor volume on the images (volume of interest (VOI) less than 100 pixels (3) secondary ovarian tumors (4) preoperative chemo- therapy (5) no pathology results |  |
|  | **Number** | 82 |  |
| Model | **Imaging technique** | axial T1-weighted nonfat suppression sequence, axial T2-weighted fast spin echo sequence, axial T2-weighted fat-suppressed turbo spin-echo sequence, coronal and sagittal T2-weighted fast spin echo sequence,  axial diffusion-weighted imaging (DWI) sequence, DCE-MRI-gadodiamode injection |  |
|  | **CAD model** | Random forest (RF) PK model = pharmacokinetic model |  |
|  | **Features** | - 6 classes of radiomics features were extracted by using pyradiomics from 7 image types from the DCE-MRI: 14 shape features, 18 first-order statistical features, 22-Gy-level cooccurrence matrix (GLCM) features, 16-Gy-level run length matrix (GLRLM) features, 16-Gy-level size zone matrix (GLSZM) features, 14-Gy-level dependence matrix (GLDM) features, 2 image filters: the Laplacian and the Gaussian filter.  - Per task radiomic features were selected: Task A 24 Task B 23 Task C 17  - A 10-fold cross-validated recursive feature elimination based on tree bagging was performed on the training cohort  - 7 features for the RF MJM model was built |  |
|  | **Test(s) used to select significant features** | (1) Mann- Whitney U (MW) test (2) the Pearson correlation coefficients (3) multivariate logistic regression backward stepwise selection |  |
|  | **Evaluation setup** | All tasks cross-validation repeated 50 times  Within the 3-class classification task, a training set (N = 72) - 70%, validation set (N=32) - 30% 1 internal validation set |  |
|  | **ROI (region of interest) annotated** | Manually by radiologist |  |
| Results | **Samples (n)** | 104  33 benign  18 borderline  53 malignant |  |
|  | **Age (years)** | 45.07 (16.21) - benign  44.56 (16.43) - borderline  51.61 (14.23) - malignant |  |
|  | **CA125 (U/ML)** | 9.46 (5.59, 26.15) - benign  14.63 (9.33, 35.22) - borderline  75.85 (14.25, 251.95) - malignant |  |
|  | **Menopausal status** | Post-menopausal  12% (40.0) - benign  7% (43.8) - borderline  20% (55.6) - malignant |  |
|  | **Timeframe scanning for surgery** | 2 weeks |  |
|  | **Timeframe data collection** | Jul 2017 - Dec 2018 |  |
|  | **Outcome** | Accuracy  Sensitivity  Specificity |  |
|  | **Intervention compared to** | Histology and radiologists |  |
| **Jian *et al.* 2021 (#11)^59^** | | | |
| **Title** MRI-Based Multiple Instance Convolutional Neural Network for Increased Accuracy in the Differentiation of Borderline and Malignant Epithelial Ovarian Tumors**.** | | |  |
| **Objective** (1) To develop an MRI- based MICNN for the differentiation of BEOT and MEOT  (2) To compare the diagnostic performance of the MICNN with that of radiologists to assess its clinical feasibility. | | |  |
| Methods | **Country** | China |  |
|  | **Funding** | Key-Area Research and Development Program of Guangdong Province, Key Research and Development Program of Jiangsu Province, Shanghai Municipal Com-mission of Health, Science and Technology Plan Projects of Jiangsu, Suzhou Science and Technology Plan Project, Shanghai Municipal Commission of Science and Technology, Shanghai Jinshan District Health Commission |  |
|  | **Design** | Retrospective case control |  |
| Participants | **Population description** | See inclusion and exclusion criteria |  |
|  | **Inclusion criteria** | (1) BEOT or MEOT proven by surgery and histopathology (2) MRI performed within 2 weeks before operation |  |
|  | **Exclusion criteria** | (1) History of gynecological operations or chemotherapy prior to the MRI scan (2) Poor quality images |  |
|  | **Number** | 501 |  |
| Model | **Imaging technique** | three axial MRI sequences: 1. fast spin echo T2-weighted imaging with fat saturation (T2WI FS) 2. echo planar diffusion-weighted imaging(DWI) with apparent diffusion coefficient (ADC) maps generated 3. 2D volumetric interpolated breath hold examination of contrast-enhanced T1-weighted imaging with FS in the late phase |  |
|  | **CAD model** | MICNN Multiple instance convolutional neural network |  |
|  | **Features** | - 3 MRI sequences (T2WI FS, DWI [with ADC map] and CE-T1WI) are most frequently used to construct our models.  - For each patient, the ADC map and CE- T1WI were aligned to T2WI FS so that the individual’s MRI data had a uniform size and spatial resolution.  - DICOM minimum and maximum applied to set pixel value to 0-1 to alleviate the effects introduced by different scanning parameters.  - Scaled, cropped and flipped preprocessing Nx244x244, was mainly composed of three modules: (A) feature extraction module for extraction of abundant disease-related features (B) multiple instance aggregation module for aggregation of features from different slices, and (C) classification module for final model decision.  - Each training (N = 342) or validation (N= 159) sample was in the form of bags. The MRI scan of each patient was treated as a bag, and the slices in each scan were referred to as instances.  512 features resulted from the bags. |  |
|  | **Test(s) used to select significant features** | N/A |  |
|  | **Evaluation setup** | Training set (N = 342) 68%, external validation set (N= 159) 32% External validation set |  |
|  | **ROI (region of interest) annotated** | Automatically |  |
| Results | **Samples (n)** | 501  borderline 165  malignant 336 |  |
|  | **Age (years)** | Mean  47.7 SD 13.7 (training)  51.6 SD 14.4 (validation) |  |
|  | **CA125 (U/ML)** | N/A |  |
|  | **Menopausal status** | N/A |  |
|  | **Timeframe scanning for surgery** | 2 weeks |  |
|  | **Timeframe data collection** | Jan 2010 - June 2018 |  |
|  | **Outcome** | Accuracy  AUC  Sensitivity  Specificity  F1 score |  |
|  | **Intervention compared to** | Histology and radiologist |  |
| **Jian *et al.* 2021 (#6)^62^** | | | |
| **Title** Multiple instance convolutional neural network with modality-based attention and contextual multi-instance learning pooling layer for effective differentiation between borderline and malignant epithelial ovarian tumors | | |  |
| **Objective** (1) a novel contextual MPL (C-MPL) that uses contextual information between adjacent instances, distinguishing it from previous permutation-invariant MPLs;  (2) a modality-based attention module for the fusion of multiple MRI modalities;  (3) to apply the C-MPL and MA module and construct a MAC- Net for BEOT/MEOT differentiation | | |  |
| Methods | **Country** | China |  |
|  | **Funding** | Key-Area Research and Development Program of Guangdong Province grants |  |
|  | **Design** | Retrospective case control study |  |
| Participants | **Population description** | See inclusion and exclusion criteria |  |
|  | **Inclusion criteria** | (1) BEOT or MEOT that was proven by surgery and histopathology (2) an MRI performed within 2 weeks before gynecological operation which included the following three axial MRI sequences, T2WI FS, ADC and CET1WI |  |
|  | **Exclusion criteria** | (1) Patients with poor quality images (2) history of gynecological operations or chemotherapy prior to the MRI scan |  |
|  | **Number** | 501 |  |
| Model | **Imaging technique** | (1) T2-weighted imaging with fat saturation (T2WI FS) (2) apparent diffusion coefficient (ADC) map, 3. contrast-enhanced T1-weighted image in the late phase (CE-T1WI). |  |
|  | **CAD model** | MAC-net Multiple instance convolutional neural network (MICNN) |  |
|  | **Features** | - A pre-trained SquzzeNet Adaptive moment estimation (Adam) with default parameters (β1 = 0.9, β2 = 0.999) was used as the optimizer.  - 20% of the training set was separated to determine the optimal hyperparameters.  - R value = 3 (neural units in intermediate layer) c = 5 |  |
|  | **Test(s) used to select significant features** | Weight-sharing |  |
|  | **Evaluation setup** | - Test set (N=382) 76% (76 hyperparameter selection)  Validation set (N=119) 23% 1 internal validation set |  |
|  | **ROI (region of interest) annotated** | Automatically |  |
| Results | **Samples (n)** | 501  336 malignant  165 borderline  7659 images per modality = 22977 images in total  Per patient 3 - 39 slices  Mean 15.29 SD 7.44 |  |
|  | **Age (years)** | N/A |  |
|  | **CA125 (U/ML)** | N/A |  |
|  | **Menopausal status** | N/A |  |
|  | **Timeframe scanning for surgery** | 2 weeks |  |
|  | **Timeframe data collection** | Jan 2010 - Jun 2018 |  |
|  | **Outcome** | Accuracy  AUC  F1 score |  |
|  | **Intervention compared to** | Histology |  |
| **Li *et al.* 2020 (#1)^61^** | | | |
| **Title** MRI-Based Machine Learning for Differentiating Borderline From Malignant Epithelial Ovarian Tumors: A Multicenter Study | | |  |
| **Objective** (1) Developed and (2) Validated an assessment model built by MRI-based machine learning for differentiating BEOT from MEOT. Diagnostic performance of the models was assessed for both whole tumor (WT) and solid tumor (ST) components | | |  |
| Methods | **Country** | China |  |
|  | **Funding** | National Natural Science Foundation of China Shanghai Municipal Commission of Science and Technology Key R&D Program of Jiangsu Foundation of Jinshan Hospital, Fudan University |  |
|  | **Design** | Retrospective case control |  |
| Participants | **Population description** | See inclusion and exclusion criteria |  |
|  | **Inclusion criteria** | 1) histologically-proven BEOT or MEOT 2) MRI performed within 2 weeks prior to gynecological operation 3) presence one of these four axial MRI sequences: 4) no chemoradiation or surgery before MRI-scanning |  |
|  | **Exclusion criteria** | 1) lacking any one of these four axial MRI sequences 2) prior gynecological operation and/or chemotherapy before MRI scanning; 3) poor MR image quality with artifacts that affected the delineation of the tumor. |  |
|  | **Number** | 501 |  |
| Model | **Imaging technique** | T2-weighted imaging (T2WI), fat saturation (FS),diffusion-weighted imaging (DWI), apparent diffusion coefficient (ADC), and contrast-enhanced (CE)-T1WI |  |
|  | **CAD model** | MP-ST (solid tumor)  CE-T1W1 (whole tumor) |  |
|  | **Features** | - For each patient, the whole tumor (WT) and the solid component of the tumor (ST) were identified on the T2WI FS sequence.  - Feature extracting using Python (Pyradiomics)  - Multiple MRI features were extracted from the WT and ST components and were subdivided into eight classes. 14 shapes 18 first order 24 GLCM 16 GLSZM 16 GLRLM 18 NGTDM 14 GLDM 744 WT.  In total 865 features from 4 MRI-sequences (T2WI, FS, DWI, ADC, and (CE)-T1WI) were divided over 8 classes  - For solid tumors (ST) and whole tumors (WT) signatures and models were built whether or not with clinical parameters (e.g. Age (years)) and incorporating the 4 MRI sequences  - In total, 10 signatures and 10 models were built, including four single-parameter signatures and corresponding models (T2WI FS, DWI, ADC, and CE-T1WI), and one multiparameter signature and corresponding model for both WT and ST features: MP signature, MP model, T2WI signature, T2WI model, FS signature, FS model, DWI signature, DWI model, ADC signature, ADC model, CE-T1WI signature, CE-T1WI model. |  |
|  | **Test(s) used to select significant features** | ICCs  Pearson correlation  matrix MRMR |  |
|  | **Evaluation setup** | - Training set (N= 250) 68% (centers A-B).  Internal validation cohort (N=92) 37%(centers A-B)  External validation (N=159) (centers C-H)  - 6 radiologists (1) 3 residents (2-3-5 years experience) (2) 3 attendings (10-12-13 years experience) 1 internal validation set  1 external validation set |  |
|  | **ROI (region of interest) annotated** | Manually by radiologist |  |
| Results | **Samples (n)** | 501  165 borderline  336 malignant |  |
|  | **Age (years)** | Mean  Training set 47.2  Internal validation 48.98  External validation 51.62 |  |
|  | **CA125 (U/ML)** | N/A |  |
|  | **Menopausal status** | N/A |  |
|  | **Timeframe scanning for surgery** | 2 weeks |  |
|  | **Timeframe data collection** | Jan 2010 - Jun 2018 |  |
|  | **Outcome** | AUC  F1 score |  |
|  | **Intervention compared to** | Histology and radiologists  (6 radiologists (1) 3 residents (2-3-5 years experience) (2) 3 attendings (10-12-13 years experience) |  |
| **Zhang *et al.* 2019 (#1)^60^** | | | |
| **Title** Magnetic resonance imaging radiomics in categorizing ovarian masses and predicting clinical outcome: a preliminary study | | |  |
| **Objective** (1) To evaluate the diagnostic performance of the MRI radiomics model in discriminating benign ovarian tumors from malignancies (2) To establish a model to validate whether MRI radiomics could differentiate between type I and type II OEC and then to assess the correlation between MRI radiomics results and histological findings (3) To a survival analysis to determine whether MRI radiomics features could determine patient prognosis | | |  |
| Methods | **Country** | China |  |
|  | **Funding** | Shanghai Emerging Advanced Technology Joint Research Project |  |
|  | **Design** | Retrospective case control study |  |
| Participants | **Population description** | Ovarian epithelial cancer |  |
|  | **Inclusion criteria** | (1) no previous pelvic surgery (2) no previous gynecological disease history (3) MRI examinations before pelvic or laparoscopic surgery were performed at our institution |  |
|  | **Exclusion criteria** | (1) previous pelvic surgical history or radiation history (2) MRI data were unavailable either for the examination performed at another institution or due to claustrophobia (3) no histological results |  |
|  | **Number** | 280 |  |
| Model | **Imaging technique** | MRI |  |
|  | **CAD model** | SVM radiomics (b-m)  SVM radiomics (I-II) |  |
|  | **Features** | - Features from 4 groups (1) intensity 22 (2) shape 15 (3) texture 39 (4) wavelets  - Total 1714 features selected eventually for all the 3 tasks |  |
|  | **Test(s) used to select significant features** | Iterative sparse representation (ISR)  Sparse representation coefficient (SRC) |  |
|  | **Evaluation setup** | - Leave-one-out cross validation (LOO) (N = 195)  - Independent testing cohort (N = 85) 1 test set |  |
|  | **ROI (region of interest) annotated** | Manually by radiologist |  |
| Results | **Samples (n)** | 280  72 benign ovarian etiologies  100 type I EOC  81 type II EOC  27 other ovarian malignancies |  |
|  | **Age (years)** | Mean Age (years) 52.7 ± 12.3 years |  |
|  | **CA125 (U/ML)** | N/A |  |
|  | **Menopausal status** | N/A |  |
|  | **Timeframe scanning for surgery** | N/A |  |
|  | **Timeframe data collection** | Jan 2014 - Dec 2017 |  |
|  | **Outcome** | Accuracy  AUC  Sensitivity  Specificity  PPV  NPV  TP rate  FP rate  TN rate  FN rate |  |
|  | **Intervention compared to** | Histology and radiologists |  |
